# Dynamic hydrogel–metal–organic framework system promotes bone regeneration in periodontitis through controlled drug delivery

**DOI:** 10.1186/s12951-024-02555-9

**Published:** 2024-05-26

**Authors:** Qipei Luo, Yuxin Yang, Chingchun Ho, Zongtai Li, Weicheng Chiu, Anqi Li, Yulin Dai, Weichang Li, Xinchun Zhang

**Affiliations:** 1 Hospital of Stomatology, Guanghua School of Stomatology, Sun Yat-sen University, No. 56, Lingyuan West Road, Guangzhou, 510055 People’s Republic of China; 2 Guangdong Provincial Key Laboratory of Stomatology, Guangzhou, 510055 People’s Republic of China

**Keywords:** Metal-organic frameworks, Hydrogel, Drug delivery, Periodontitis, Bone regeneration

## Abstract

Periodontitis is a prevalent chronic inflammatory disease, which leads to gradual degradation of alveolar bone. The challenges persist in achieving effective alveolar bone repair due to the unique bacterial microenvironment’s impact on immune responses. This study explores a novel approach utilizing Metal–Organic Frameworks (MOFs) (comprising magnesium and gallic acid) for promoting bone regeneration in periodontitis, which focuses on the physiological roles of magnesium ions in bone repair and gallic acid's antioxidant and immunomodulatory properties. However, the dynamic oral environment and irregular periodontal pockets pose challenges for sustained drug delivery. A smart responsive hydrogel system, integrating Carboxymethyl Chitosan (CMCS), Dextran (DEX) and 4-formylphenylboronic acid (4-FPBA) was designed to address this problem. The injectable self-healing hydrogel forms a dual-crosslinked network, incorporating the MOF and rendering its on-demand release sensitive to reactive oxygen species (ROS) levels and pH levels of periodontitis. We seek to analyze the hydrogel’s synergistic effects with MOFs in antibacterial functions, immunomodulation and promotion of bone regeneration in periodontitis. In vivo and in vitro experiment validated the system's efficacy in inhibiting inflammation-related genes and proteins expression to foster periodontal bone regeneration. This dynamic hydrogel system with MOFs, shows promise as a potential therapeutic avenue for addressing the challenges in bone regeneration in periodontitis.

**Graphical Abstract:**

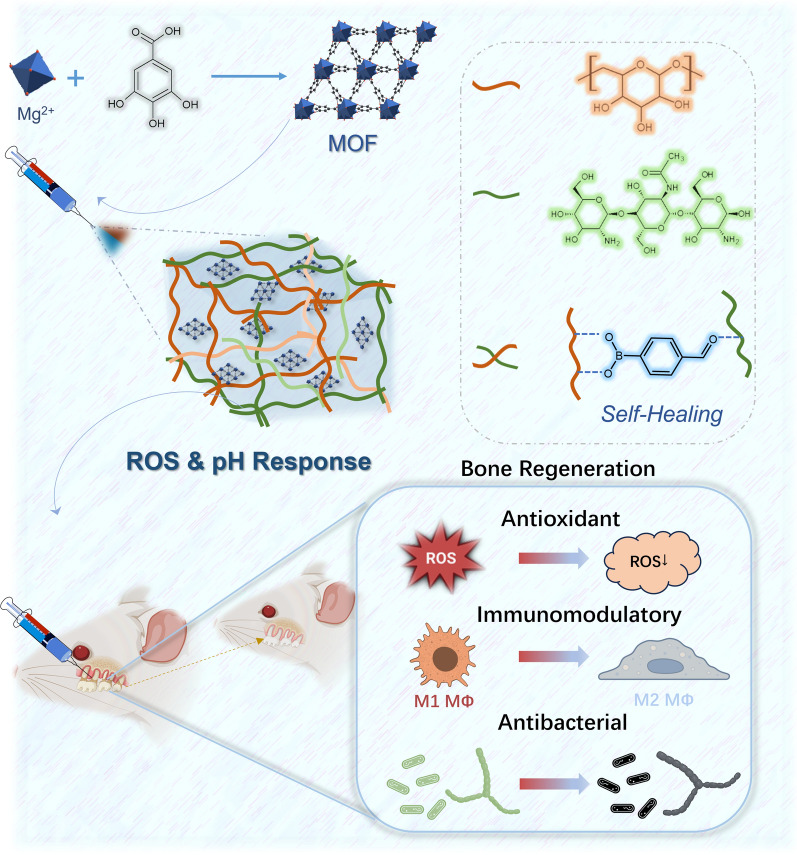

**Supplementary Information:**

The online version contains supplementary material available at 10.1186/s12951-024-02555-9.

## Introduction

Periodontitis is a chronic inflammatory disease initiated by dental plaque, characterized with periodontal soft tissue inflammation and progressive destruction of the periodontal ligament and alveolar bone [1]. Periodontitis is one of the most common oral diseases globally, affecting approximately 740 million individuals [2]. Currently, the established treatment strategy for periodontitis involves the removal of causative factors through methods such as scaling and root planning [3]. Guided bone regeneration (GBR) surgical procedures are commonly employed for alveolar bone regeneration in the context of periodontitis [4]. Throughout the progression of periodontitis, *Porphyromonas gingivalis* (*P. gingivalis*) is recognized as the primary pathogen, residing in periodontal pockets and creating anaerobic and alkaline environments [5, 6]. Within this unique bacterial microenvironment of periodontitis, immune cells produce excessive pro-inflammatory cytokines and reactive oxygen species (ROS), consequently leading to hinder the bone regeneration process [7, 8]. During the process, the significantly higher ratio of M1/M2 macrophages interacts with excessive ROS deposit and inflammatory microenvironment causing uncontrollable periodontal tissue damage [9]. Nowadays, multiple biomaterials have been developed for the treatment of periodontitis, including antibacterial agents, ROS-scavenging and Immunomodulatory biomaterials [10, 11]. However, most biomaterials and traditional treatment approaches performed single function, failing to meet the multifunctional requirements for bone regeneration in periodontitis for infection caused by the colonization of pathogens at the defect site is one of the main causes of GBR failure as reported [12, 13], thus, there is an urgent need for an effective treatment modality for treating periodontitis.

Based on the physiological characteristics of periodontitis [5–8], effective treatment necessitates functions such as antimicrobial activity, antioxidant property and pro-osteogenesis capacity. Generally, various classes of antibiotics are utilized to achieve the aforementioned functions [14]. However, the widespread misuse of these drugs may inevitably lead to drawbacks such as drug resistance and adverse side effects [15]. Therefore, researchers are gradually shifting their focus towards the exploration of novel therapeutic agents. In recent years, the application of Metal–Organic Frameworks (MOFs) in the field of biomedicine, particularly in drug delivery and disease treatment, has garnered widespread attention [16]. MOFs are a class of porous crystalline materials composed of metal ions and organic linkers, characterized by customizable components and structures, enabling the design and development of multifunctional systems tailored to various diseases [17]. It has been reported that Mg^2+^, a cation present in cellular environments and second only to potassium ions in concentration, plays a crucial role in various physiological functions, including energy metabolism, protein synthesis and cellular signaling [18, 19]. Appropriate concentrations of magnesium ions have been shown to not only directly promote bone repair to some extent [20], but also guide macrophages to polarize from the M1 phenotype to the M2 phenotype, thereby creating an immunomicroenvironment conducive to bone formation [21]. On the other hand, gallic acid (GA), a natural secondary metabolite rich in phenolic hydroxyl groups that effectively scavenges reactive oxygen species (ROS) and disrupts the cyclic generation of new free radicals [22], can regulate the bone regeneration process by its antioxidant properties [23, 24]. Furthermore, GA exerts anti-inflammatory effects by reducing the release of pro-inflammatory cytokines and chemokines [25]. Therefore, MOFs constructed from magnesium and GA (Mg-GA) have the potential advantage for the treatment of bone defects induced by periodontitis.

However, during the treatment of periodontitis, the dynamic and complex oral environment, including factors such as saliva flow, chemical composition and temperature fluctuations, can lead to rapid loss of MOFs drugs [26]. Additionally, the irregular anatomical structures of periodontal pockets further present potential challenges in the treatment of periodontitis [27]. Therefore, there is a need to develop a sustained release system for controlling the on-demand release of MOFs and providing a favorable microenvironment for bone regeneration in periodontitis. In recent years, injectable hydrogel materials have gradually gained attention in the biomedical field [28–30], especially smart responsive hydrogels, which offer unique advantages in periodontitis treatment [31, 32]. Hence, this study aims to construct a dynamic responsive smart injectable hydrogel in conjunction with MOFs of Mg-GA for the treatment of periodontitis. Initially, we have selected Carboxymethyl Chitosan (CMCS) and Dextran (DEX) as the primary components for constructing the injectable hydrogel. Carboxymethyl chitosan (CMCS), a derivative of chitosan with similar biocompatibility and enhanced water solubility and antibacterial property [33], is widely used in tissue engineering, anti-bacterial dressing and drug delivery system [34]. As well as Dextran (DEX) is a kind of linear polysaccharide compound produced by bacteria *Leuconostoc mesenteroides* [35]. The backbone of DEX is linked with α-1,6-glycosidic bond occasionally branched with α-1,2-, α-1,3- and α-1,4-glycosidic bonds thus rich in hydroxyl groups [36]. Though related to dental plaque formation [37], based on DEX's excellent water solubility, biocompatibility, biodegradability, cell adhesion properties and anti-inflammatory effects, it has been extensively studied in biomedical delivery systems [38]. However, relying solely on the weak hydrogen bonding between CMCS and DEX, it is challenging to form a stable hydrogel structure [39, 40].

The utilization of covalent bonds to construct a dual-crosslinked network significantly enhances the mechanical stability of hydrogels [41, 42]. In this study, we employed 4-formylphenylboronic acid (4-FPBA) as a crosslinking agent, which can form dynamic borate ester bonds and imine bonds (Schiff base) with dextran (DEX) and carboxymethyl chitosan (CMCS), respectively. The *in-situ* formation of an interpenetrating network structure (CMCS/4-FPBA/DEX, CSBDX) under the influence of 4-FPBA not only enhances the mechanical strength of the hydrogel but also imparts injectable properties to it [43]. When applied in the treatment of periodontitis, the hydrogel can be conveniently injected and filled into deep periodontal pockets [44]. Moreover, in the dynamic and complex oral environment, the self-healing properties of the hydrogel maintain long-term stability [43]. Interestingly, the imine bonds and borate ester bonds within the hydrogel network exhibit pH and ROS sensitivity, respectively [44, 45]. The high pH and ROS levels in the microenvironment of periodontitis can influence the three-dimensional network structure of the hydrogel, thereby actively regulating on-demand drug release.

**Fig. 1:**
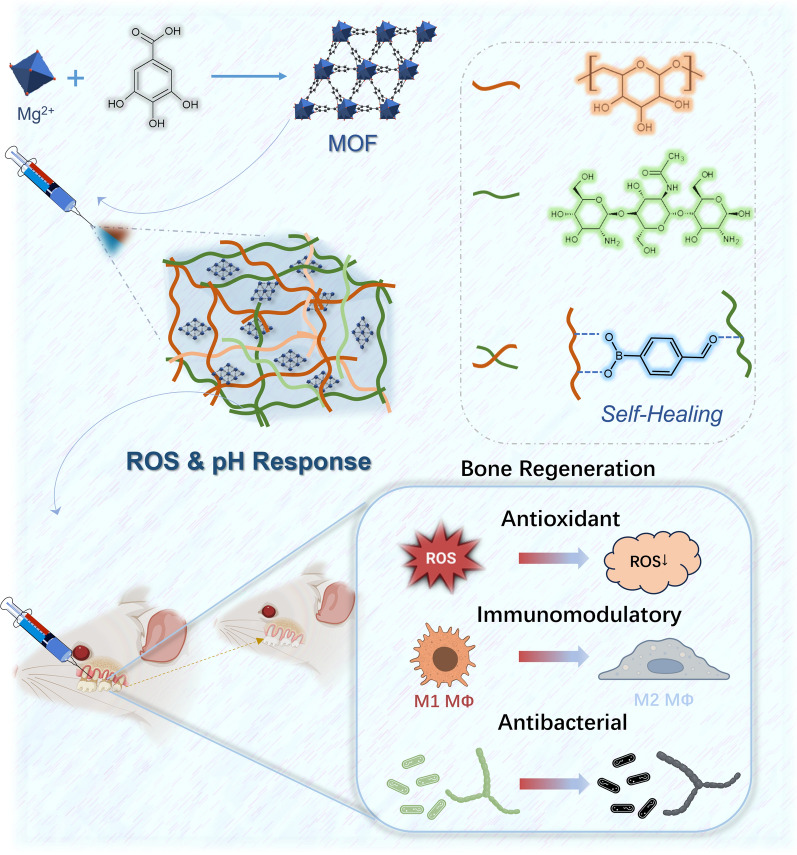
The schematic illustration of the preparation, application and bone regeneration promotion mechanism of CSBDX@MOF

In this study, the MOF of Mg-GA was loaded into CSBDX injectable self-healing hydrogels for the treatment of periodontitis (Fig. 1). We aim to investigate in-depth the on-demand release of Mg-GA under the specific microenvironment stimuli of periodontitis, by modulating its impact on osteogenesis through the regulation of oxidative stress and the immune microenvironment. The study aims to elucidate the synergistic effects of the hydrogel's self-healing properties and antibacterial functions in conjunction with the MOF. Furthermore, we intend to validate the inhibitory effects on inflammation and promotion of alveolar bone regeneration of the CSBDX@MOF system through in vivo periodontitis model experiments. Our hydrogel system holds the potential to offer a novel approach for bone regeneration in periodontitis.

## Methods and experiments

### Materials

Magnesium chloride (99.99%), gallic acid (99%) and KOH (99.99%) were purchased from Macklin (Shanghai, China). Carboxymethyl chitosan was purchased from Xiya Reagent (Shandong, China). 4-formylphenylboronic acid was purchased from Adamas-beta (Shanghai, China). Dextran (M.W. 150,000) were purchased from J&K Scientific (Beijing, China). RAW264.7 and MC3T3-E1 cells were acquired from the Shanghai Cell Bank of the Chinese Academy of Sciences (Shanghai, China). Lipopolysaccharide from *Porphyromonas gingivalis* was purchased from InvivoGen (USA). DPPH (2,2-Diphenyl-1-picrylhydrazyl) and ABTS [2,2'-Azinobis-(3-ethylbenzthiazoline-6-sulphonate)] were purchased from Aladdin Chemical Technology (Shanghai, China). South American fetal bovine serum, α-MEM culture medium, DMEM culture medium, penicillin, Alexa Fluor 488 Phalloidin, DAPI, LIVE/DEAD Bac Light and anti-OCN antibody were purchased from Thermo-Fisher Scientific (USA). DCFH-DA and BCIP/NBT Alkaline Phosphatase Color Development Kit were purchased from Beyotime Biotechnology (China). 3.7 wt% paraformaldehyde and yeast extract were purchased from Biosharp Biotechnology (China). β-streptomycin, sodium glycerophosphate, dexamethasone, vitamin C, 1% Triton X-100, bovine serum albumin and phosphate buffer solution were purchased from Sigma-Aldrich (China). Prime-script RT reagent Kit, and SYBR Premix EX Taq was purchased from TaKaRa Biotechnology (Japan). Cell Counting Kit-8 was purchased from DOJINDO laboratories (Japan). Calcein-AM/propidium iodide (PI) double staining kit was purchased from Bestbio (China). Nutrient Agar, L-cysteine hydrochloride, hematin chloride, vitamin K1 and Alizarin Red S Solution (1%, pH = 4.2) were purchased from Solarbio LIFE SCIENCES (China). Brain Heart Infusion culture media was purchased from HuanKai Microbial (China).

### Synthesis of Mg-GA

Biocompatible and non-toxic MOF of Mg-GA was synthesized using a simple hydrothermal method. In brief, MgCl_2_ (1 g) and gallic acid (3.8 g) were dissolved in ultrapure water (50 mL). Then, the solution pH was adjusted to 8 with potassium hydroxide solution (10 mol/L). The milkly white solution gradually turned to brown. After stirring for 10 min, the solution was moved to a 100 mL autoclave and heated at 120 ℃ for 24 h. The solution was cooled to room temperature, centrifuged at 10,000 rpm for 10 min, and the supernatant discarded. The light grey solid pellet was obtained by washing three times with ultrapure water and dried in an oven at 60 ℃ overnight.

### Synthesis of the hydrogels

For the preparation of Carboxymethyl Chitosan (CMCS)/Dextran (DEX)/4-formylphenylboronic acid (4-FPBA)/Mg-GA composite hydrogels, 4.8 wt% 4-FPBA and 2 wt% DEX were first dissolved in double distilled water. Then, the Mg-GA powders were dissolved in 4 wt % CMCS solution at a concentration of 0.5 wt%, 1 wt% and 2 wt%. Equal volumes of the two solutions were homogenously mixed and quickly injected into the mold and hydrogels were formed within minutes. CMCS/4-FPBA/DEX hydrogels were obtained in the same way as described above without Mg-GA. Hereafter, in this paper, the hydrogels loaded with Mg-GA will be denoted as CSBDX@MOF, which is divided into CSBDX@2.5MOF, CSBDX@5MOF, and CSBDX@10MOF according to different mass ratios from low Mg-GA composition to high composition (2.5 mg/mL, 5 mg/mL, 10 mg/mL). Then, the hydrogels without Mg-GA will be represented as CSBDX.

### Characterization of CSBDX

The chemical compositions of lyophilized CSBDX samples were analyzed by Fourier transform infrared (FTIR). Injectability property of the CSBDX was observed visually by loading the hydrogel into a 1 mL syringe and injecting it manually to write words ‘SYSU’. After minutes of gelation, photographs were taken. The self-healing property of the CSBDX was evaluated by cutting the hydrogel in half, one of which was colored by crystal violet, and simply put them together to ensure the cross-sections completely adhered. After 24 h at 37 ℃, the self-healing hydrogel was picked up by tweezer remaining intact. The self-healing interface of another piece of self-healing hydrogel was observed by stereomicroscope. Rheological continuous step strain test was performed to further reveal the self-healing behavior of CSBDX hydrogel. The CSBDX samples with a diameter of about 25 mm and a thickness of 1 mm were prepared and HAAKE MARS Modular Advanced Rheometer System (Thermo Scientific) was used to carry out continuous step strain test at 37 ℃. The parameters were set: angular frequency of 6.28 rad/s, strain 1% (duration 100 s) → 100% (100 s) → 1% (100 s) → 100% (100 s) → 1% (100 s).

### Characterization of Mg-GA

The morphology of the Mg-GA was observed by scanning electron microscopy (SEM). The crystal structure of Mg-GA was examined by X-ray diffraction (XRD). The elemental composition of Mg-GA was measured by energy dispersive spectroscopy (EDS) coupled with SEM and X-ray photoelectron spectroscopy (XPS).

### Characterization of CSBDX@MOF

#### Surface and cross-section morphology

The surface roughness of the membranes was observed using a laser scanning confocal microscope profilometer (LSM700, Zeiss, Germany). Sa (the arithmetic average height deviation from the mean plane) was selected to describe the surface roughness of hydrogels. The water contact angle was measured using a DSA-X ROLL contact angle measuring instrument. The profile of a drop of 2 μL of deionized water on the surface of hydrogels was captured. The intersection angles of both sides were measured to calculate average values. SEM and EDS were used to observe the cross-section morphology and elemental composition of lyophilized hydrogels. All samples were sputter-coated with gold layers and then analyzed using a Zeiss Sigma 300 field-emission Scanning Electron Microscope. The obtained SEM images were evaluated using Image J software to analyze the average pore diameters. Meanwhile, scanning maps of B, C, O, N, Mg were also measured by EDS.

#### Mechanical properties

Mechanical properties of hydrogels were tested using a universal testing machine (Instron 5967, USA). For tensile testing, cuboid hydrogel samples 2 mm in width and 1mm in thickness were clamped on tensile grips probe, the initial distance between the grips was 6mm. The samples were stretched at a speed of 1 mm/min until breakage. The tensile mechanical evaluation index included the stress–strain curve, largest tensile strength and elongation at break. For compression testing, cylinder hydrogel samples 10 mm in diameter and 5 mm in thickness were placed between two compression plates and compressed at a rate of 1  mm/min. Compressive modulus was determined from the obtained stress–strain curves. For cyclic compression testing, hydrogel samples of same size were compressed at a rate of 1  mm/min until strain reached 50%, then the compression was released and the entire cycle was repeated 5 times.

#### Swelling and degradation properties

The following methods were used to detect the swelling properties of hydrogels in vitro: The initial mass of the lyophilized hydrogel was weighed as W_0_. Then the samples were immersed in phosphate buffer saline (PBS) at 7.4 pH and 37 ℃. At different time intervals (1 h, 3 h, 6 h, 12 h,24 h,48 h), the mass of the swollen gel was measured as W_d_ after the removal of extra water on the gel with filter paper. The swelling ratio (SR) of the hydrogel was defined by formula (1).

1 $${\text{Swelling Ratio }}\,\left( \% \right) = {{{\text{W}}_{{\text{d}}} } \mathord{\left/ {\vphantom {{{\text{W}}_{{\text{d}}} } {{\text{W}}_{0} }}} \right. \kern-0pt} {{\text{W}}_{0} }} \times 100\%$$

As to degradation properties of hydrogels, swollen hydrogels were immersed in 10 mL phosphate buffer saline (PBS) at 7.4 pH and 37 ℃. The initial mass of the lyophilized hydrogel was weighed as W_0._ At each time point (1st, 4rd, 7th, 14th, 21st, 28th day), the samples were removed, dried gently with filter paper and then weighed as W_d_. The remaining weight was calculated using the following formula (2).

2 $${\text{Remaining Weight }}\,\left( \% \right) = {{{\text{W}}_{{\text{d}}} } \mathord{\left/ {\vphantom {{{\text{W}}_{{\text{d}}} } {{\text{W}}_{0} }}} \right. \kern-0pt} {{\text{W}}_{0} }} \times 100\%$$

#### Drug release properties

CSBDX@10MOF was selected to evaluate the responsive and sustained release performance. Hydrogel samples were prepared and placed in centrifuge tube. 20 mL PBS with pH values of 7.40 and 9.00 and 1 mM H_2_O_2_ was added into the centrifuge tube and placed at 37 ℃. After the scheduled time, 1 μL of the solution was removed and the absorption intensity at 260 nm (characteristic peak of Mg-GA [66]) was analyzed by Spectrophotometer (Thermo Nanodrop One, USA).

#### Antibacterial test

*Porphyromonas gingivalis* (*P. gingivalis*, ATCC 33277) and *Actinobacillus actinomycetemcomitans* (*A. a.*, ATCC 43717) were selected to test the antibacterial activity of hydrogels. First, CSBDX and CSBDX@MOF were fabricated as described above and sterilized by placing under UV light for 1 h. Then, the hydrogel samples were immersed in bacterial suspension (1.0 × 10^6^ CFU/mL, 3 mL) at concentration of 0.2 g/mL in 5 mL centrifuge tubes and incubated in an anaerobic environment at 37 ℃ for 24 h. For observation of the colony number after culture, the bacterial suspension was diluted, and spread evenly onto Columbia Blood Agar Plates (*P. gingivalis*) or BHI Agar Plates (*A. a.*). The plates were incubated in an anaerobic environment at 37 °C, and the colonies were observed and calculated. For Live/Dead bacterial staining, the hydrogel samples were centrifuged and washed 3 times with PBS to remove excess medium and then stained using a Live/Dead Bac Light bacterial viability kits according to the manufacturer's protocol. The fluorescence images were taken by laser scanning confocal microscopy. To observe the morphology of *P. gingivalis* and *A. a.*, the hydrogel samples were first centrifuged and washed 3 times with PBS. Then, the hydrogel samples were fixed with 2.5% glutaraldehyde, dehydrated using increasing concentrations of ethanol (30, 50, 70, 80, 90 and 100% v/v) and lyophilized. After that, the samples were placed on silicon wafers and sputter-coated with gold layers. The bacterial morphology was observed using a Zeiss Sigma 300 field-emission Scanning Electron Microscope.

### Biocompatibility

#### Preparation of hydrogel extracts

Hydrogel extracts were prepared according to national standards GB/T 16886.12. In brief, hydrogel was prepared and immersed in DMEM or α-MEM medium (0.2 g/mL) at 37 ℃ for 72 h. The medium was then centrifuged to acquire the hydrogel extracts, and the extracts were stored at 2 ~ 8 ℃ for further experiments.

#### Cell proliferation assay

RAW 264.7 and MC3T3 cells were seeded in a 48 well-plate at a density of 1 × 10^5^ cells per well and cultured with hydrogel extracts. On the 1st, 3rd, and 5th day, cell proliferation was evaluated using the CCK-8 kit. Briefly, the working solution was added and incubated for 2 h in the dark at 37 ℃ and 5% CO_2_. The optical density (OD) was measured at 450 nm using a microplate reader (Thermo 3001, USA).

#### Live/dead cell staining

The cell viability on the 1st, 3rd, and 5th day was evaluated using a calcein-AM/PI double stain kit. After RAW 264.7 and MC3T3 cells cultured with hydrogel extracts and rinsed twice with PBS, a mixed solution of 1 μM calcein-AM and 3 μM PI was added. After incubation in the dark for 30 min, the cells were observed using a fluorescence microscope (Zeiss Axio Vert. A1, Oberkochen, Germany).

#### Cell morphology observation

To observe morphology of the cells cultured with hydrogel extracts, the cells were incubated for 24 h, washed twice with PBS, and stained with Alexa Fluor 488 Phalloidin and DAPI. The cells were then observed using a laser scanning confocal microscope (Olympus FV3000, Japan).

#### Hemolysis assays

Hemolysis induced by hydrogels was tested using 2% rabbit red blood cells (Sbjbio life sciences, Nanjing, China). In brief, 1mL of the RBCs were incubated with hydrogel at 37 ℃ for 1 h. The mixtures were then centrifuged at RCF of 1000 g for 10 min. Hemoglobin absorption at 576 nm (OD_576 sample_) of each supernatant (150 µL) was measured. Hemolysis activity was then determined according to the following formula (3).

3 $$\text{H}{\text{emolysis}}\left(\text{\%}\right)\text{=}\left[\left({\text{OD}}_{\text{576 sample}}-{\text{OD}}_{\text{576 blank}}\right)\text{/(}{\text{OD}}_{\text{576 Triton}}-{\text{OD}}_{\text{576 blank}}\text{)}\right]$$

PBS (OD_576 blank_) was used as a reference for 0% hemolysis, while the absorbance (OD_576 Triton_) of RBCs incubated with 0.1% Triton-X 100 solution was set as 100% hemolysis.

### Antioxidant property

#### Total antioxidant activity measurement

The radical scavenging performance was tested using the DPPH/ABTS method. In brief, hydrogel samples of 50 mg were incubated with 3 mL 0.2 mM DPPH/ethanol solution in the dark at RT for 30 min. The supernatant of the solution was removed into 96 well-plate and the scavenging activity was evaluated by monitoring the absorbance decrease at 517 nm. DPPH radical scavenging activity was calculated according to the following formula (4).

4 $${\text{DPPH scavenging rate}} = \left( {1 - \frac{{A_{1} - A_{2} }}{{A_{0} }}} \right){ } \times {\text{100\% }}$$

where A_0_ is the absorbance of the control (the well base of the plate), A_1_ is the absorbance of samples, and A_2_ is the absorbance of samples incubated in ethanol instead of DPPH solution during incubation.

The ABTS•^+^ scavenging activity was determined by spectrophotometric analysis with the characteristic absorption at 734 nm. The ABTS•^+^ was produced by mixing 2 mL of ABTS (7.00mM) with 2 mL of K_2_S_2_O_8_ (4.95 mM) in the dark at 25 ℃ for 12 h. Dilute the solution to a working solution of 5% concentration with PBS (0.1 mM) to make the absorbance of the solution at 734 nm was about 0.7. ABTS•^+^ solution (3 mL) was incubated with hydrogel samples of 50 mg for 30 min, and the supernatant was monitored by a plate reader. ABTS radical scavenging activity was calculated according to the following formula (5).

5 $${\text{ABTS scavenging rate}} = \left( {1 - \frac{{A_{1} - A_{2} }}{{A_{0} }}} \right){ } \times {\text{100\% }}$$

where A_0_ is the absorbance of the control (the well base of the plate), A_1_ is the absorbance of samples, and A_2_ is the absorbance of samples incubated in PBS instead of ABTS solution during incubation.

#### Intracellular ROS scavenging measurement

RAW 264.7 cells were seeded in a 48 well-plate at a density of 1 × 10^5^ cells per well and cultured with hydrogel extracts for 24 h. Then, the culture medium was replaced with H_2_O_2_-containing culture medium (300 μM) and incubated for 1 h. Subsequently, cells were stained with 2,7-dichlorofluorescein diacetate (DCFH-DA) to label intracellular ROS fluorescently and imaged using a fluorescence microscope (Zeiss Axio Vert. A1, Oberkochen, Germany); the ROS ( +) reagent in the kit was used as a positive control. The fluorescence intensity was then quantitated with Multi-Detection Reader (Biotek-Synergy H1, USA). To further evaluate the ROS scavenging effect of hydrogel extracts quantitatively and accurately in vitro, After the above treatment, RAW 264.7 cells were detached and the intracellular ROS level was determined through fluorescence intensity with flow cytometry (BD LSRFortessa, USA).

### Immunomodulatory property

The anti-inflammatory capacity was evaluated in an *P. gingivalis*-LPS-stimulated macrophage model. In this part, the preparation of hydrogel extracts was the same as that in “Biocompatibility” part. RAW264.7 cells were stimulated with *P. gingivalis*-LPS (1 μg/mL) for 24 h to simulate inflammation in periodontitis. For L/CSBDX, L/CSBDX@2.5MOF, L/CSBDX@5MOF and L/CSBDX@10MOF groups, the macrophages were treated with corresponding hydrogel extracts and *P. gingivalis*-LPS (1 μg/mL) for 24 h. Quantitative polymerase chain reaction (qPCR) was used to detect the expression of inflammatory and anti-inflammatory genes. The total cellular RNA was extracted using the RNA-Quick Purification Kit. RNA was reverse transcribed into cDNA using the PrimeScriptTM RT reagent kit. The real-time qPCR system (ABI QuantStudio 5, USA) was used to detect the expression of iNOS, COX-2, IL-6, TGF-β3, IL-10 and CD206 genes, which were standardized by the housekeeping gene β-actin. Relative gene expression was compared using the 2^−ΔΔCt^ method. Table S1 shows the primer sequences used in this study. The expression of iNOS and CD206 protein in macrophages was observed by immunofluorescence staining. The samples were fixed in 4% paraformaldehyde for 15 min and permeabilized with 0.1% TritonX-100 for 20min. Samples were further incubated with anti-CD206 antibody (Cell Signaling Technology, #24,595) or anti-iNOS antibody (Cell Signaling Technology, #13,120) overnight, followed by incubation with the corresponding fluorescently labeled secondary antibody for 1 h and DAPI for 15 min, and the cells were imaged by laser scanning confocal microscope (Olympus FV3000, Japan). The macrophage polarization surface markers (CD86 and CD206) were detected by flow cytometry. Briefly, RAW264.7 cells were collected and washed three times with PBS. Then, cells were incubated with antibodies (FITC anti-mouse CD86 and PE anti-mouse CD206 (both from Biolegend)) on ice for 30 min in the dark. After washed with PBS twice, cells were suspended in 500 μl PBS with 3% FBS and then detected by flow cytometry (BD Biosciences, San Diego, CA, USA).

### Osteogenic differentiation in vitro

Macrophage conditioned medium (MCM) was used to investigate whether hydrogel extracts could affect the osteogenic differentiation of MC3T3 cells through the immunomodulatory effect of macrophages. For MCM preparation, RAW 264.7 cells were treated with different hydrogel extracts (CSBDX, CSBDX@2.5MOF, CSBDX@5MOF and CSBDX@10MOF) and *P. gingivalis*-LPS for 24 h. Then, the supernatants were collected and centrifuged at 1000 rpm for 10 min to remove remained cells. After that, the supernatants were mixed with fresh α-MEM medium at a ratio of 1:2 and then filtered with a 0.22 μm filter to obtain MCM. MC3T3 cells were seeded at a density of 1 × 10^5^ cells per well in α-MEM medium for 12 h, then the medium was replaced by MCM for 24 h. After osteogenic induction (10 mM β-glycerophosphate, 0.1 μM dexamethasone and 50 mM ascorbate) for 7 days, ALP staining was performed. After osteogenic induction for 7 days and 14 days, quantitative polymerase chain reaction (qPCR) was used as above to detect the expression of osteogenic-related genes (RUNX2, ALP, COL1, OCN, OPN and OPG). Table S1 shows the primer sequences used in this study. Besides, the expression of RUNX2 and OCN protein was observed by Immunofluorescence staining. The operation steps were the same as above except using anti-RUNX2 antibody (Cell Signaling Technology, #12,556) and anti-OCN antibody (Thermo-fisher, PA5-96,529). After osteogenic induction for 21 days, the ECM mineralization was evaluated by the Alizarin Red S Staining.

### Animal experiments

#### Rat periodontitis model establishment

Twenty-four male Sprague Dawley rats (weight 200 ± 20 g) were purchased from the Experimental Animal Center of Sun Yat-sen University. All the experimental procedures were complied with the NIH Guide for Care and Use of Laboratory Animals. All the experimental procedures were approved by Animal Ethics Committee of Sun Yat-sen University (Approval No. SYSU-IACUC-2022-002019) and animal experiments were conducted in the Animal Centre of Sun Yat-sen University. Animals were randomly divided into four groups (for each group, N = 6), including healthy rats without periodontitis (CON), periodontitis without any treatment (PD), periodontitis treated with CSBDX (CSBDX), periodontitis treated with CSBDX@10MOF (CSBDX@MOF). To establish the periodontitis model, all Sprague Dawley rats were first anesthetized with pentobarbital sodium (40 mg/kg body weight, Sigma, USA). Next, a 4–0 surgical suture was wrapped and fixed around the second maxillary molar for 10 days. After the periodontitis model was established, the surgical sutures and food residues were removed. Subgingival scaling and root planning were performed using gracey and then 10 μL of hydrogels (CSBDX and CSBDX@10MOF) were injected once into the periodontal pockets with a microsyringe. For the PD group, 10 μL of PBS was injected as negative control. The CON group acted as a blank control without any treatment. On the 7th day after treatment, half the rats were anesthetized with pentobarbital sodium and sacrificed and on the 28th day, the other half were sacrificed. Then, the maxillae were collected and fixed in 4% PFA for 1 day and then preserved in 70% alcohol at 4 ℃ for further experiments.

#### Micro-CT analysis

Micro-CT was adopted to analyze bone regeneration in vivo using a micro-CT scanner (µCT 50 cabinet microCT scanner, SCANCO Medical AG, Bassersdorf, Zurich, Switzerland). Dissected rat maxillae were horizontally placed into the sample tubes, and parameters were set: 55 kVp, 177 mA, and 10 μm resolution to start the scan. The three-dimensional (3D) reconstruction was performed using Amira-Avizo software (Version 2020.1, Thermo Scientific). The reconstructed images was captured by complying with the identical criteria that all the tooth cusps were located on the same plane, and the occlusion plane could not be seen from the buccal side or the palatal side. The vertical distance between the cementoenamel junction (CEJ) and the alveolar bone crest (ABC), namely CEJ–AB, was measured, representing the degree of alveolar bone loss. The ratio of bone volume/tissue volume (BV/TV) of each sample was also calculated.

#### Histological analysis

The fixed samples were decalcified with 10% disodium ethylenediamine tetraacetate (EDTA) for 4 weeks. Afterward, the specimens were dehydrated in a graded series of ethanol solutions. After the transparent treatment with xylene, the specimen was embedded in paraffin to obtain paraffin sections with a thickness of 5 μm. Hematoxylin and eosin (H&E) and Masson staining was performed to assess periodontal tissue regeneration. Immunohistochemical staining for iNOS and CD206 was performed to assess the immunomodulatory effects and immunohistochemical staining for COL1 (anti-COL1 antibody, Cell Signaling Technology, #72,026) and OCN were performed to assess the osteogenic effects. The staining of specimens was observed under a microscope and the positive cells per high power field (HPF) were calculated.

### Statistical analysis

The sample size for each statistical analysis was n ≥ 3. All data were presented as the mean ± standard deviation (SD) of at least three independent experiments. Data normality was assessed by the Shapiro–Wilk test. For data normally distributed, one-way ANOVA combined with Bonferroni’s test was performed to analyze the group-to-group significant differences in the SPSS software (SPSS 20.0). The nonparametric tests were performed when the data were not normally distributed. A *p* value of less than 0.05 was considered to be statistically significant (Fig. 1).

## Results and discussion

### Preparation and characterization of Mg-GA and CSBDX

**Fig. 2:**
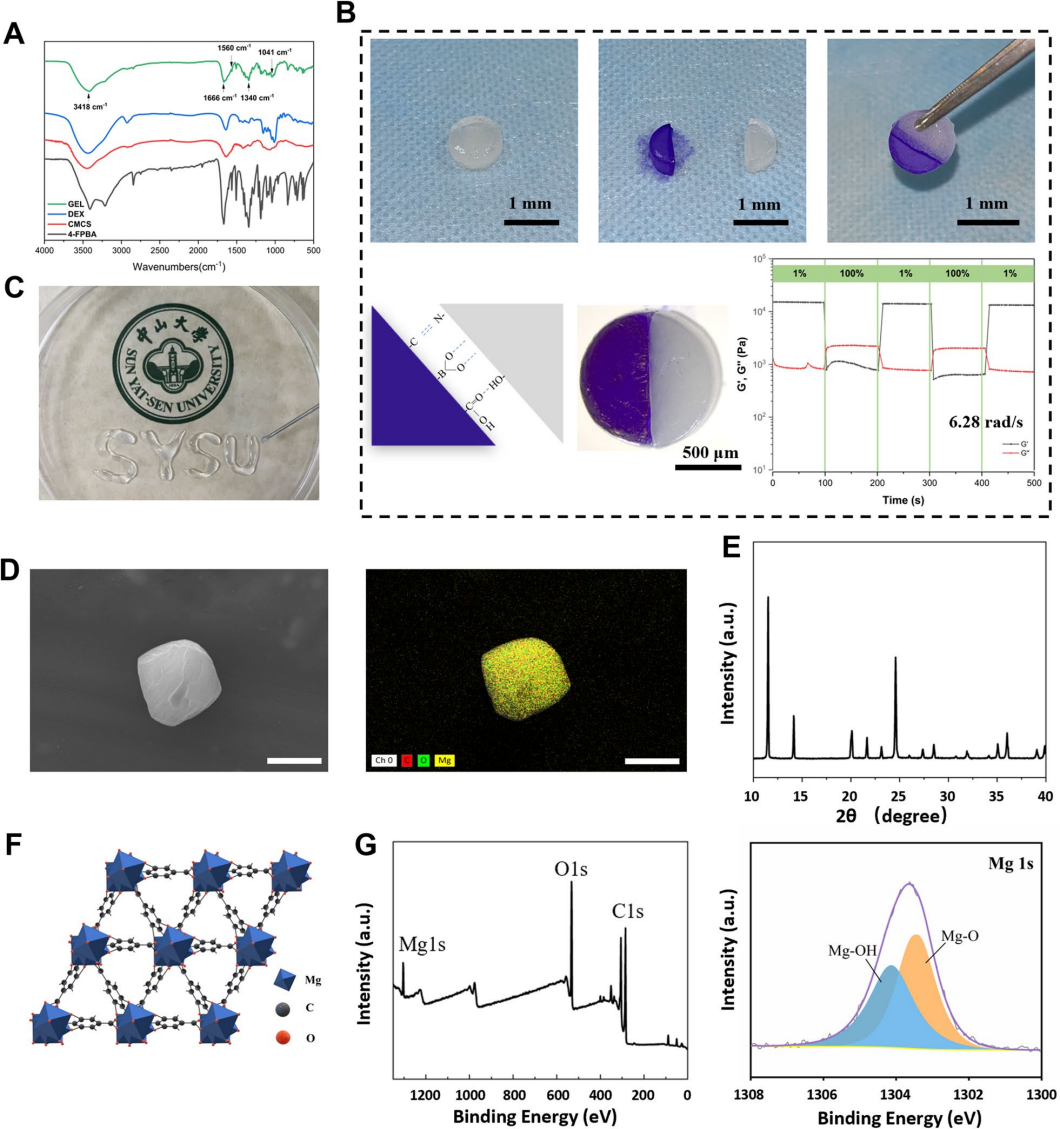
The characterization of CSBDX and Mg-GA. **A** The FT-IR spectrum of CSBDX and each component. **B** Self-healing property of CSBDX. Two pieces of self-healed hydrogel can support its own weight and formation of dynamic Schiff base and boronic ester bond and self-healing interface was observed with microscope and continuous step-strain measurement. **C** The injectability of CSBDX. **D** The morphology and EDS mapping images of Mg-GA (bar = 2 μm). **E** The XRD spectrum of Mg-GA. **F** The structure schematic diagram of Mg-GA. **G** The XPS spectrum of Mg-GA and peak-differentiating and imitating of Mg element

The synthesis of CMCS/4-FPBA/DEX (CSBDX) was investigated by a simple mixing method in situ. FT-IR spectra of various components was shown in Fig. 2A. Specifically, the major peaks occurred at 3418 cm^–1^ (O–H stretching vibration of hydroxyl and phenolic hydroxyl group), 1666 cm^–1^ (C = O stretching vibration of amide I and carbonyl group), 1340 cm^–1^ (B-O stretching vibration) and 1041 cm^–1^ (C–O–C antisymmetric stretching vibration of cyclic ether group of DEX). It is noteworthy that C = N stretching vibration of Schiff’s base bond which usually occurred at 1660–1620 cm^–1^ [46] was shifted to the newly formed peak at 1560 cm^–1^. These results demonstrated successful introduction of borate ester bond and Schiff’s base bond into CSBDX. After incubating the incised hydrogel at 37 °C for a period of time, the hydrogel undergoes self-healing in the absence of external conditions, with no noticeable gaps observed at the interface after healing. This outcome can be attributed to the collaborative action of dynamic borate ester bonds, Schiff base bonds and hydrogen bonds within the hydrogel network (Fig. 2B). Under rheological step strain test, a large strain higher than tensile strain at break of CSBDX was applied to evaluate self-healing property of CSBDX after destruction. The results showed that the mechanical property of CSBDX was well restored after repeated 100% strain with the G’ and G’’ slightly decreased. The self-healing properties of the hydrogel enable it to adapt to the complex and dynamic oral environment during application. Furthermore, the hydrogel exhibited excellent injectability, smoothly extruding through a medical syringe and quickly forming a gel, making it well-suited for the environment of deep periodontal pockets in the treatment of periodontitis (Fig. 2C). The hydrogel system, designed with dynamic chemical bonds, combines injectability and self-healing properties effectively, thus meeting the application requirements for periodontitis treatment [47, 48]. MOFs of Mg-GA were successfully synthesized using the classical hydrothermal method. Microscopic analysis of their morphology and elemental composition (Fig. 2D, Fig. S1A) revealed that Mg-GA exhibits an irregular framework shape with a diameter of approximately 1 μm as well as the homogeneous distribution of Mg, C and O elements within Mg-GA particles. X-ray diffraction (XRD) spectra yielded results were consistent with previous reports [49], revealing that each Mg atom was connected to organic ligands of GA by six O atoms (four oxygen atoms coming from the phenol groups and two from the carboxyl functions) and the pristine crystal structure of Mg-MOF was maintained (Fig. 2E**, **Fig. 2F). Furthermore, X-ray photoelectron spectroscopy (XPS) was employed to verify the elemental composition of Mg-GA, confirming the presence of Mg, C and O elements, consistent with the EDS results. Additionally, the peak-differentiating and imitating of Mg element suggested that the construction of Mg-GA was primarily achieved through the coordination between Mg^2+^ and hydroxyl or carbonyl groups (Fig. 2G). The above results showed the successful synthesis of Mg-GA by verifying the morphology, elemental composition and stable crystal structure of Mg-GA as well as the intrinsic connecting structure between Mg^2+^ and GA.

### Preparation and characterization of CSBDX@MOF

**Fig. 3:**
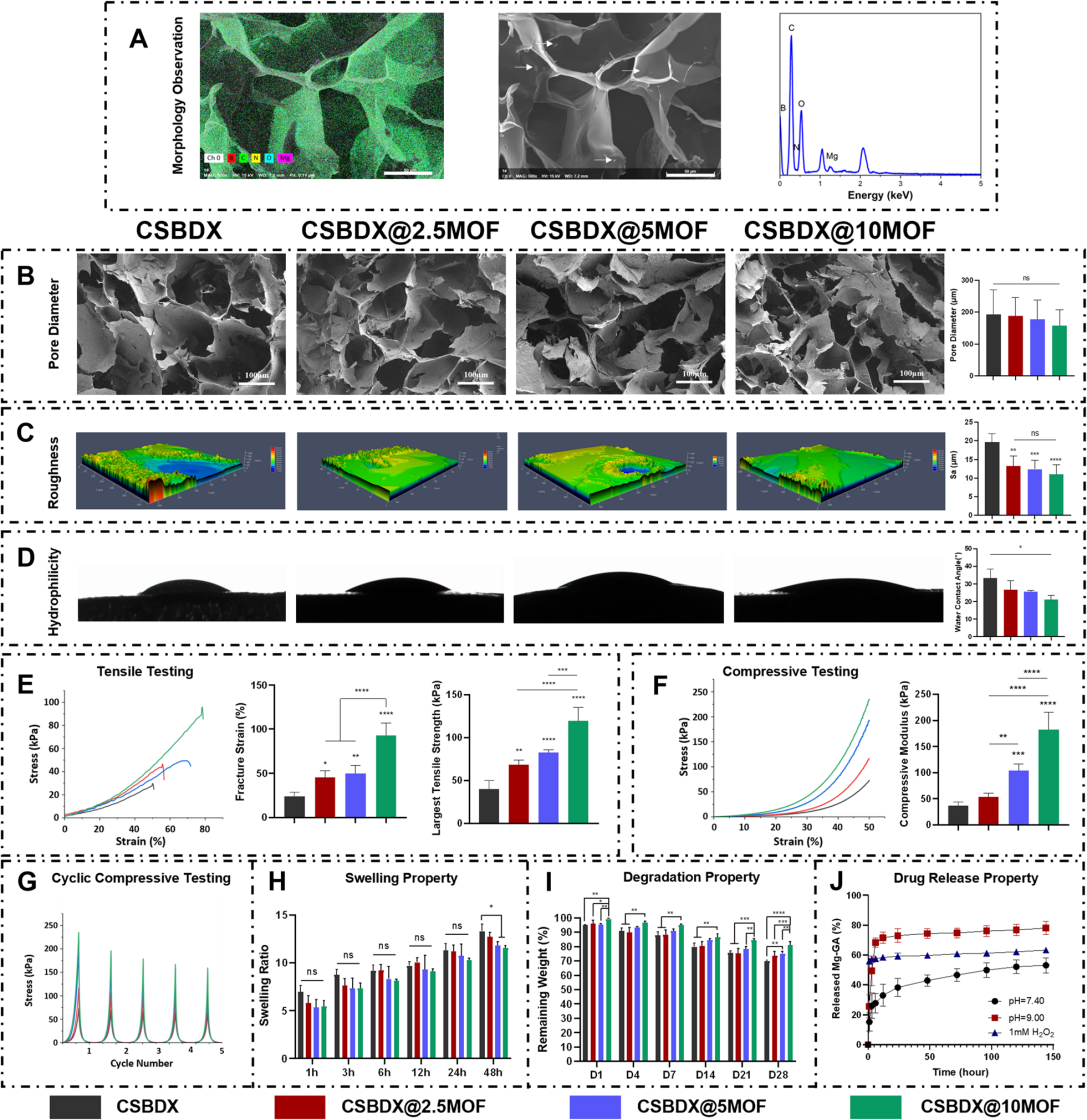
The characterization of CSBDX@MOF. **A** The morphology, EDS mapping images and energy spectrum of CSBDX@5MOF. The white indicates Mg-GA particles (bar = 50 μm). **B** The cross-sectional morphology and pore diameter histogram of CSBDX@MOF (bar = 100 μm, n = 6). **C** The visualizations of surface morphology and Sa (the arithmetic average height deviation from a mean plane) values of different surfaces of CSBDX@MOF (n = 6). **D** The water contact angles of CSBDX@MOF (n = 3). **E** The representative tensile stress–strain curves, fracture strain and largest tensile strength of CSBDX@MOF (n = 6). **F** The representative compressive stress–strain curves and compressive modulus of CSBDX@MOF (n = 6). **G** The cyclic compressive stress–strain curves of CSBDX@MOF (n = 6). The swelling profile **H** and degradation profile **I** of CSBDX@MOF (n = 4). **J** The Mg-GA releasing profile of CSBDX@10MOF in different condition. *: *P* < 0.05, **: *P* < 0.01, ***: *P* < 0.001, ****: *P* < 0.0001, ns: no significance

To further investigate the influence of different concentrations of Mg-GA MOF on the properties and functionalities of hydrogels, composite hydrogels with varying Mg-GA content (2.5 mg/mL, 5 mg/mL, 10 mg/mL) were prepared. These hydrogels were abbreviated as CSBDX@2.5MOF, CSBDX@5MOF and CSBDX@10MOF, respectively. SEM micrographs revealed a well-defined porous structure within the hydrogels, with micrometer-scale Mg-GA particles evenly distributed throughout the hydrogel network, as indicated by the white arrow. Complemented by EDS spectral images, the presence of B, C, N, O and Mg elements confirmed the successful incorporation of Mg-GA (Fig. 3A, Fig. S1B). The cross-sectional microstructure of the hydrogel exhibited a porous architecture with pore sizes ranging from 150 μm to 250 μm **(**Fig. 3B). Furthermore, an increase in Mg-GA concentration showed a higher presence of Mg-GA particles within the hydrogel network, without significant changes in pore size, indicating that variations in Mg-GA concentration did not affect the hydrogel’s microstructure. Notably, a pore size range of 100–300 µm is favorable for the transportation of oxygen and nutrients, promoting cellular infiltration and tissue ingrowth [50, 51]. Surface roughness and hydrophilicity of the hydrogel samples were also evaluated. Introduction of Mg-GA led to a significant decrease in the Sa value (arithmetical mean deviation of the profile) but showed no significant difference among the three CSBDX@MOF groups (Fig. 3C). As it is well-known, a rough surface favors cell adhesion, migration [52] and bacterial attachment [53]. Surfaces with relatively lower roughness, as seen in CSBDX@MOF, might reduce the adherence of periodontal pathogens, preventing persistent tissue infections. Water contact angle results indicated excellent hydrophilicity for all hydrogel samples, with a decrease in water contact angle which was attributed to the rich phenolic hydroxyl groups present in the MOF of Mg-GA [54] (Fig. 3D). As depicted in Fig. 3E, the fracture strain and maximum tensile strength of CSBDX@MOF were higher compared to CSBDX, with CSBDX@10MOF exhibiting the highest fracture strain (up to 92.97 ± 14.04%) and maximum tensile strength (up to 119.5 ± 15.72 kPa). Compressive testing showed a similar trend to the tensile results, where the introduction of Mg-GA increased the compressive modulus of the hydrogel, reaching a maximum of 182.5 ± 32.94 kPa for CSBDX@10MOF (Fig. 3F). Moreover, all hydrogels maintained their structural integrity and elasticity after 5 cycles of compression (Fig. 3G). The enhanced mechanical strength of the hydrogel system resulted from the formation of new hydrogen bonds between the hydroxyl groups of Mg-GA and CSBDX, along with the coordination interactions between a small amount of free Mg^2+^ ions and carboxyl groups of CSBDX, stabilizing the hydrogel structure. All groups exhibited a similar swelling behavior, but CSBDX@5MOF and CSBDX@10MOF displayed significantly lower swelling ratios compared to CSBDX after 48 h of swelling (Fig. 3H). This was due to the introduction of an appropriate amount of MOF, which restructured the three-dimensional network of the hydrogel, making it denser. Additionally, the degradation behavior of the hydrogel was significantly affected by the concentration of Mg-GA, with CSBDX@10MOF showing the lowest degradation (Fig. 3I). This aligns with expectations, as the introduction of Mg-GA enhanced the crosslinking and stability of the hydrogel. These results collectively indicated that CSBDX@10MOF exhibited superior hydrophilicity, mechanical performance, resistance to swelling and stability. Hence, CSBDX@10MOF is preferred for further investigations. The release behavior of Mg-GA under different conditions within the hydrogel system is depicted in Fig. 3J. The drug release curves demonstrated a typical sustained release pattern of Mg-GA in pH = 7.4 PBS, with an accumulated release of 53.13% over 144 h. In an environment simulating high ROS levels (1mM H_2_O_2_), the release efficiency significantly increased, reaching 60% within 48 h. In contrast, in a high pH environment (pH = 9.0), the release rate was slower but achieved a markedly higher maximum cumulative release of 74.43%, surpassing that in the 1mM H_2_O_2_ group. These outcomes suggested significant pH and ROS sensitivity of CSBDX@MOF, attributed to the dynamic crosslinking effects of borate ester bonds and imine bonds [44, 45]. Therefore, the drug release process is responsive to the high pH and ROS microenvironment of periodontitis, enabling on-demand release of MOF of Mg-GA, thereby facilitating the treatment process of periodontitis.

### Antibacterial effect

**Fig. 4:**
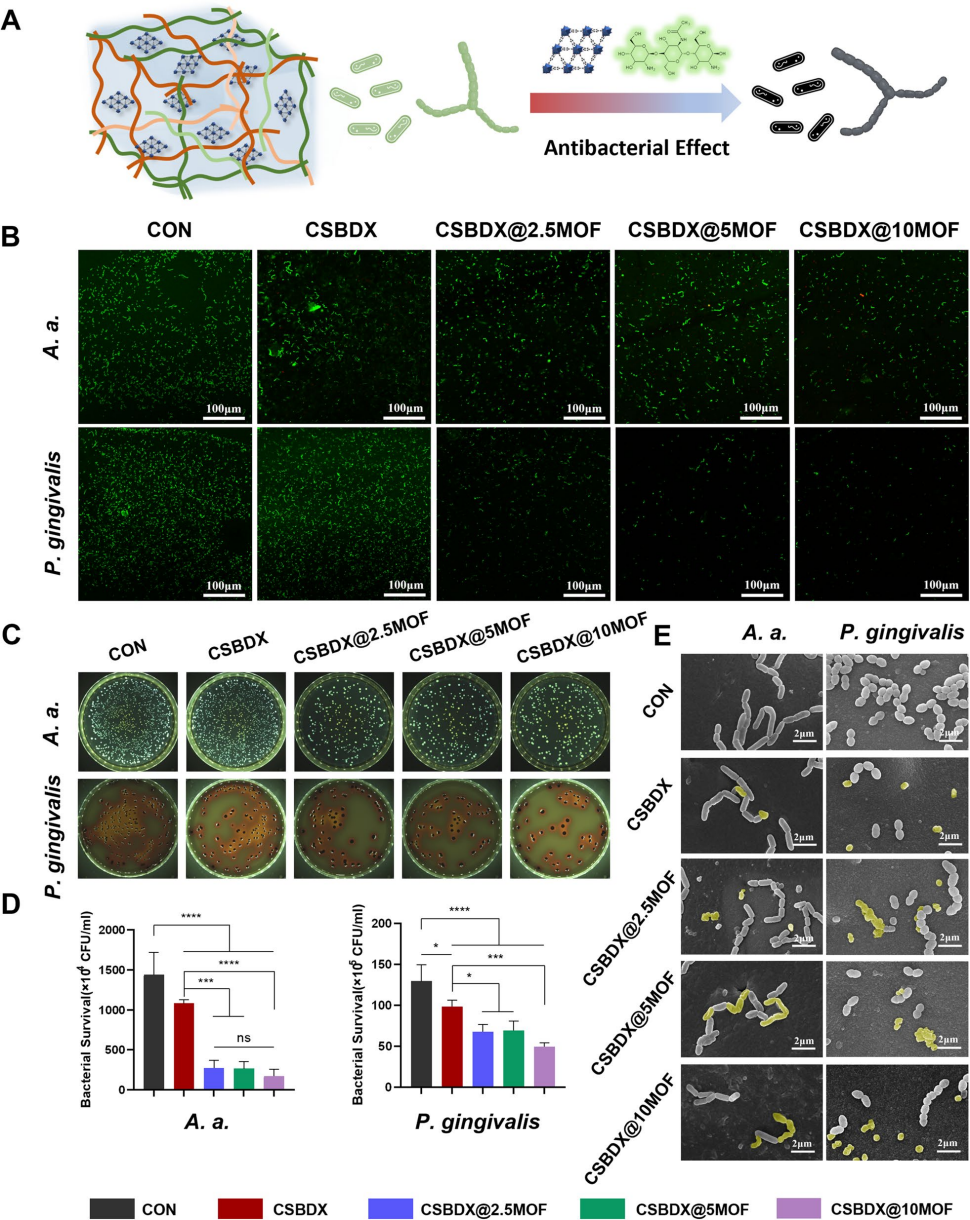
Antibacterial effect. **A** The schematic illustration of antibacterial effect of hydrogel system on *A. a.* and *P. gingivalis*. **B** Representative images of Live/dead bacteria staining of *A. a.* and *P. gingivalis* after co-culture with hydrogel (bar = 100 μm). **C** Representative images of colony formation assay and bacterial survival histogram **D** of *A. a.* and *P. gingivalis* after co-culture with hydrogel and corresponding statistical analysis of bacterial survival (n = 4). **E** Representative SEM images of *A. a.* and *P. gingivalis* after co-culture with hydrogel (bar = 2 μm). Yellow pseudo-color indicates the stacked and crumpled bacteria. *: *P* < 0.05, ***: *P* < 0.001, ****: *P* < 0.0001, *ns* no significance

The adhesion and growth of various oral bacteria directly influence the therapeutic efficacy of periodontitis [13]. Therefore, achieving antimicrobial functionality is a pivotal factor in the application of medical materials during the treatment for periodontitis. In this study, we selected *Actinobacillus actinomycetemcomitans* (*A. a.*) and *P. gingivalis* as the research targets to investigate the in vitro antibacterial effects of a hydrogel system (Fig. 4A). *A. a.* and *P. gingivalis* are major pathogenic bacteria associated with periodontitis [55], whose presence not only acts as the initiating factors of periodontitis but also directly hinders the bone regeneration process. Live/dead bacterial staining (Fig. 4B) revealed a reduced quantity of live bacteria in the CSBDX group. A few red-stained dead bacteria were observed, suggesting that CSBDX and CSBDX@MOF hydrogel systems probably play a role in inhibiting bacterial proliferation or affecting bacterial function. Colony-forming unit assays (Fig. 4C) demonstrated a significant reduction in colony formation in CSBDX@MOF groups compared to the control. Both the live bacterial count and colony-forming units in the three CSBDX@MOF groups notably decreased compared to CSBDX group. Besides, quantitative analysis of colony counts (Fig. 4D) indicated that CSBDX@10MOF exhibited the fewest colony-forming units among the CSBDX@MOF groups. Subsequently, scanning electron microscopy (SEM) was employed to further examine the microscopic morphological changes in bacteria (Fig. 4E). The results indicated a consistent decrease in the number of bacteria in the field of view with the aforementioned colony analysis. Some bacteria in the CSBDX group exhibited wrinkling, while more stacked and wrinkled bacteria were observed in the CSBDX@MOF groups. These findings suggested that both CSBDX and CSBDX@MOF could inhibit the growth of *A. a.* and *P. gingivalis*. This is attributed to the intrinsic antimicrobial functionality of the CMCS components in both groups [56], where the antibacterial mechanism of CMCS is similar to CS and arises from the electrostatic interaction between the material and bacteria [57]. The stronger antibacterial action of the CSBDX@MOF groups is attributed to the presence of MOFs of Mg-GA. MOFs may significantly enhance antibacterial functionality through synergistic effects via physical contact as reported [58]. Therefore, CSBDX@MOF exhibits superior antibacterial efficacy against periodontitis pathogens, creating a favorable microenvironment for bone regeneration in periodontitis [1].

### Biocompatibility

**Fig. 5:**
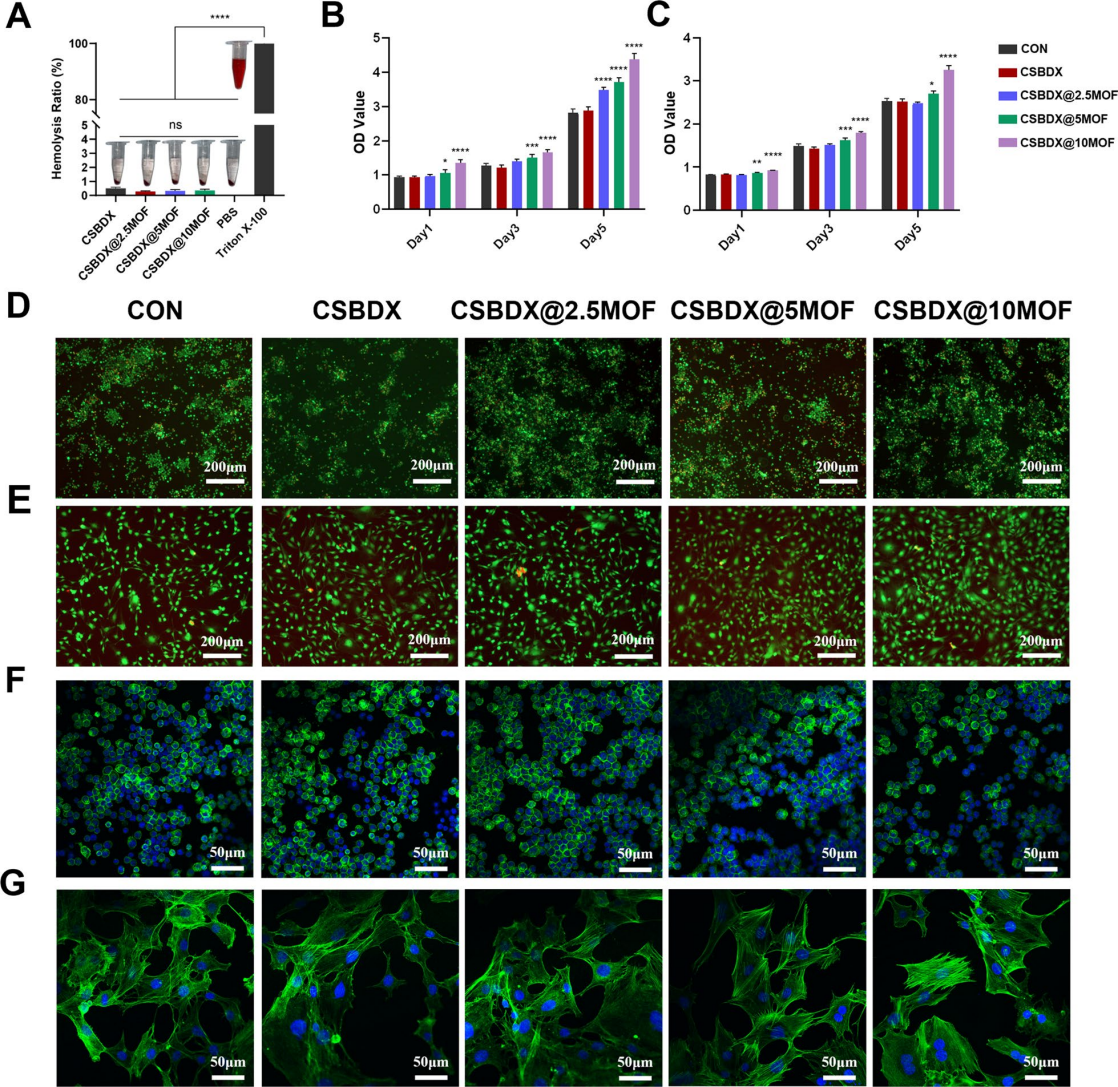
The biocompatibility of CSBDX@MOF. **A** Hemolysis ratio of rabbit erythrocytes incubated with hydrogels (n = 4). The RAW264.7s **B** and MC3T3 cells **C** proliferation quantitatively analyzed by CCK-8 after treated with hydrogel extracts for 1, 3, and 5 days (n = 4). Representative live/dead staining of RAW264.7s **E** and MC3T3 cells **E** cultured with hydrogel extracts for 5 days (bar = 200 μm). The RAW264.7s **F** and MC3T3s **G** morphology after cultured with hydrogel extracts for 5 days (bar = 50 μm). *: *P* < 0.05, **: *P* < 0.01, ***: *P* < 0.001, ****: *P* < 0.0001

Bleeding symptom is common clinical manifestation of periodontal disease and the hemocompatibility of the hydrogel system is fundamental for treating periodontitis [1]. As depicted in Fig. 5A, the hemolysis rates of all hydrogel groups (CSBDX, CSBDX@2.5MOF, CSBDX@5MOF and CSBDX@10MOF) were less than 2%, showing no significant difference compared to the PBS control group, indicating their excellent hemocompatibility. Good biocompatibility is a crucial parameter for the in vivo application of biomedical materials. In this study, RAW 264.7 and MC3T3 cells were chosen, and a classic method (GB/T 16886.12) was employed to investigate the biocompatibility of the material system. The CCK-8 assay was used to evaluate cytotoxicity (Fig. 5B, C). Interestingly, the introduction of MOFs had a certain stimulating effect on cell proliferation, especially on days 3 and 5, with the most pronounced effect observed in the CSBDX@10MOF group with the highest MOFs content. This phenomenon is attributed to the gradual release or degradation of Mg-GA, leading to the gradual release of Mg^2+^, which assists in promoting cell proliferation [59]. Live/dead staining, as shown in Fig. 5D, 5E, revealed gradual cell proliferation consistent with CCK-8 results, with only a small number of red dead cells observed (Fig. S2). Almost all cells remained viable and exhibited a green color after 5 days of culture. Cell morphology serves as one of the indicators of cellular physiological status and function [60]. To further examine the impact of the culture process on cell morphology, this study performed nuclear (blue) and actin cytoskeleton (green) staining on the cells. Results showed that RAW 264.7 cells appeared round with island-like distribution, while MC3T3 cells exhibited polygonal shapes with extended pseudopodia interconnections, similar to the control group (Fig. 5F–G), indicating that the hydrogel system did not affect cell morphology. These results demonstrated that the hydrogel system exhibited good cell compatibility and the ability to promote cell proliferation, thereby suggesting its potential role in tissue regeneration.

### Antioxidant property

**Fig. 6:**
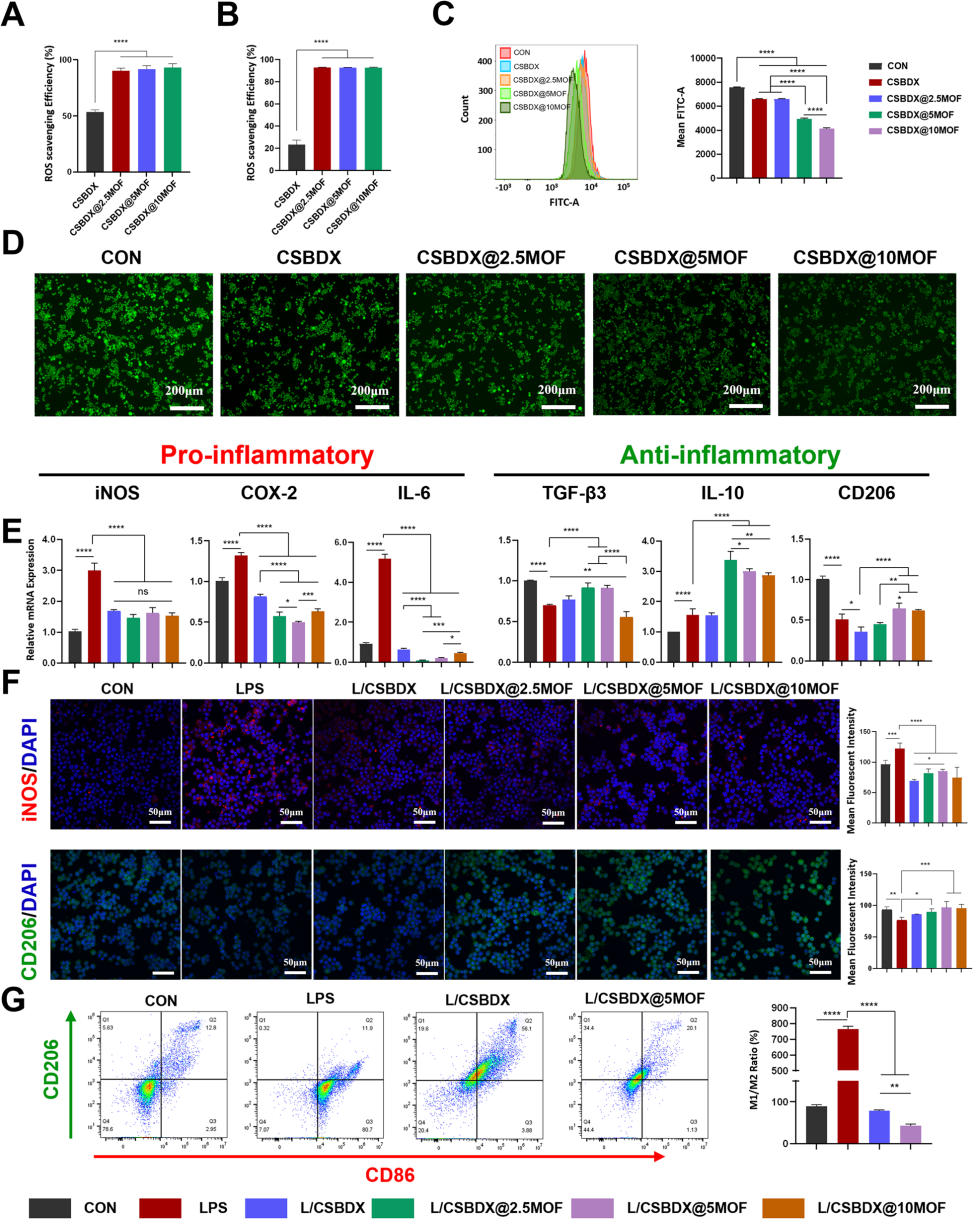
The antioxidant and immunomodulatory properties of CSBDX@MOF. The quantitative analysis of DPPH **A** and ABTS **B** radical scavenging efficiency of hydrogel. **C** Representative flow cytometry data and mean fluorescence intensity of DCF in RAW264.7 treated with 300 μM H2O2 with hydrogel extracts. **D** Representative DCFH-DA staining images of in RAW264.7 treated with 300 Μm H2O2 (bar = 200 μm). Relative gene (pro-inflammatory: iNOS, COX-2 and IL-6. Anti-inflammatory: TGF-β3, IL-10 and CD206) expressions **E** and macrophage polarization-related protein (iNOS and CD206) expressions **F** in RAW264.7 incubated with hydrogel extracts with stimulation of 1 μg/mL P.g.-LPS for 24 h (bar = 50 μm). **G** Flow cytometry of macrophage polarization surface markers (CD86 and CD206). *: P < 0.05, **: P < 0.01, ***: P < 0.001, ****: P < 0.0001

The oxidative stress microenvironment in periodontitis constitutes a pivotal impediment to repair and treatment [7]. Throughout the progression of periodontitis, the innate immune system triggered by periodontitis pathogens generates responses, including the upregulation of pro-inflammatory cytokines and reactive oxygen species (ROS) aimed at protecting the host from bacterial invasion, consequently leading to varying degrees of periodontal tissue damage [8]. Here, the practical antioxidant function of hydrogels was determined using DPPH and ABTS free radical scavenging assays. As depicted in Fig. 6A, B, CSBDX exhibited approximately 50% and 20% scavenging capacity against DPPH and ABTS free radicals, respectively. However, upon incorporation of MOFs into the hydrogel, the three different MOF content hydrogel systems exhibited free radical scavenging activities exceeding 90% for both DPPH and ABTS. The results indicated the excellent ROS scavenging ability of the hydrogel systems directly correlated with MOFs, primarily driven by the antioxidant component GA [22]. Additionally, the macrophage intracellular ROS scavenging activity of hydrogels in an environment rich in H_2_O_2_ is crucial. Flow cytometry analysis **(**Fig. 6C) demonstrated significantly lower mean fluorescence intensity (MFI) for CSBDX@5MOF and CSBDX@10MOF compared to CSBDX and CSBDX@2.5MOF, indicating higher intracellular ROS scavenging activity in hydrogel systems with higher MOF content. CSBDX@2.5MOF exhibited relatively limited intracellular ROS scavenging activity due to the lower content of Mg-GA. Consistent results were observed between fluorescence imaging and flow cytometry (Fig. 6D). These findings suggested that the hydrogel doped with MOFs of Mg-GA exhibits excellent antioxidative stress capabilities, possibly due to the hydrogen donating tendencies arising from hydroxyl, phenolic and easily ionizable carboxylic groups within the MOFs [22, 61]. The outstanding extracellular and intracellular antioxidative characteristics of CSBDX@MOF effectively mitigate oxidative stress, thereby providing favorable conditions for the improvement of the bone regeneration microenvironment in periodontitis.

### Immunomodulatory property

Reportedly, Mg-GA MOFs exhibits significant immunomodulatory effects [62], where the Mg^2+^ and GA component reduced the generation of pro-inflammatory mediators [63, 64] and increased the M2/M1 ratio [65] of macrophages to foster an immune microenvironment promoting bone regeneration. In this study, a model of inflammatory microenvironment was constructed utilizing lipopolysaccharide (LPS) derived from *P. gingivalis* to investigate the immunomodulatory effects of hydrogel systems. Stimulation with LPS notably increased the mRNA expression levels of pro-inflammatory genes (iNOS, COX-2 and IL-6) in macrophages, while exhibiting a downregulation trend in the mRNA expression levels of anti-inflammatory genes (TGF-β3, IL-10 and CD206) (Fig. 6E). Immunofluorescence staining also revealed an upregulation of iNOS protein expression and a downregulation of CD206 protein expression (Fig. 6F). Furthermore, LPS and hydrogel treatment of macrophages led to a downregulation of mRNA expression levels of pro-inflammatory genes. The hydrogel groups (L/CSBDX, L/CSBDX@2.5MOF, L/CSBDX@5MOF and L/CSBDX@10MOF) displayed iNOS mRNA expression levels trending towards the control group, with no significant differences among the hydrogel groups. The CSBDX group exhibited minimal influence on anti-inflammatory genes, while the introduction of MOFs significantly upregulated mRNA expression levels in the CSBDX@MOF group. Particularly, the upregulation of CD206 mRNA expression was notably affected by CSBDX@5MOF and CSBDX@10MOF, and CSBDX@5MOF exhibited a favorable promoting effect on the upregulation of IL-10 and TGF-β3 mRNA expressions (Fig. 6E). Validation of iNOS and CD206 protein expression levels in immunofluorescence staining corroborated these results (Fig. 6F). The cytometry results of macrophage polarization surface markers (CD86 and CD206) also verified the immunomodulatory property of CSBDX@5MOF (Fig. 6G). Given the close association between the skeletal and immune systems, alterations in the immune microenvironment influence the balance between bone resorption and regeneration [66]. During the development of periodontitis, recruited macrophages infiltrate periodontal tissues and macrophage polarization is closely associated with the pathogenesis of periodontitis [67]. Activated macrophages are known to assume two phenotypes with different functionalities (M1 and M2). Polarization of the M1 phenotype correlates with chronic inflammation and bone resorption, where iNOS is considered a marker of M1 macrophages, and M1 polarization upregulates the expression of IL-6, COX-2, iNOS, and other inflammatory mediators. M2 macrophages determine the resolution of inflammation and bone reconstruction, promoting the expression of TGF-β3 and IL-10 [68]. Based on the inherent anti-inflammatory capacity of DEX, hydrogels primarily composed of DEX exhibit the functionality of suppressing the expression of M1-related genes [69]. Therefore, the synergistic effects of the DEX component combined with the released Mg^2+^ and GA in the CSBDX@MOF system contribute to improving the immune microenvironment for bone regeneration.

### Osteogenesis property in vitro through immunomodulation

**Fig. 7:**
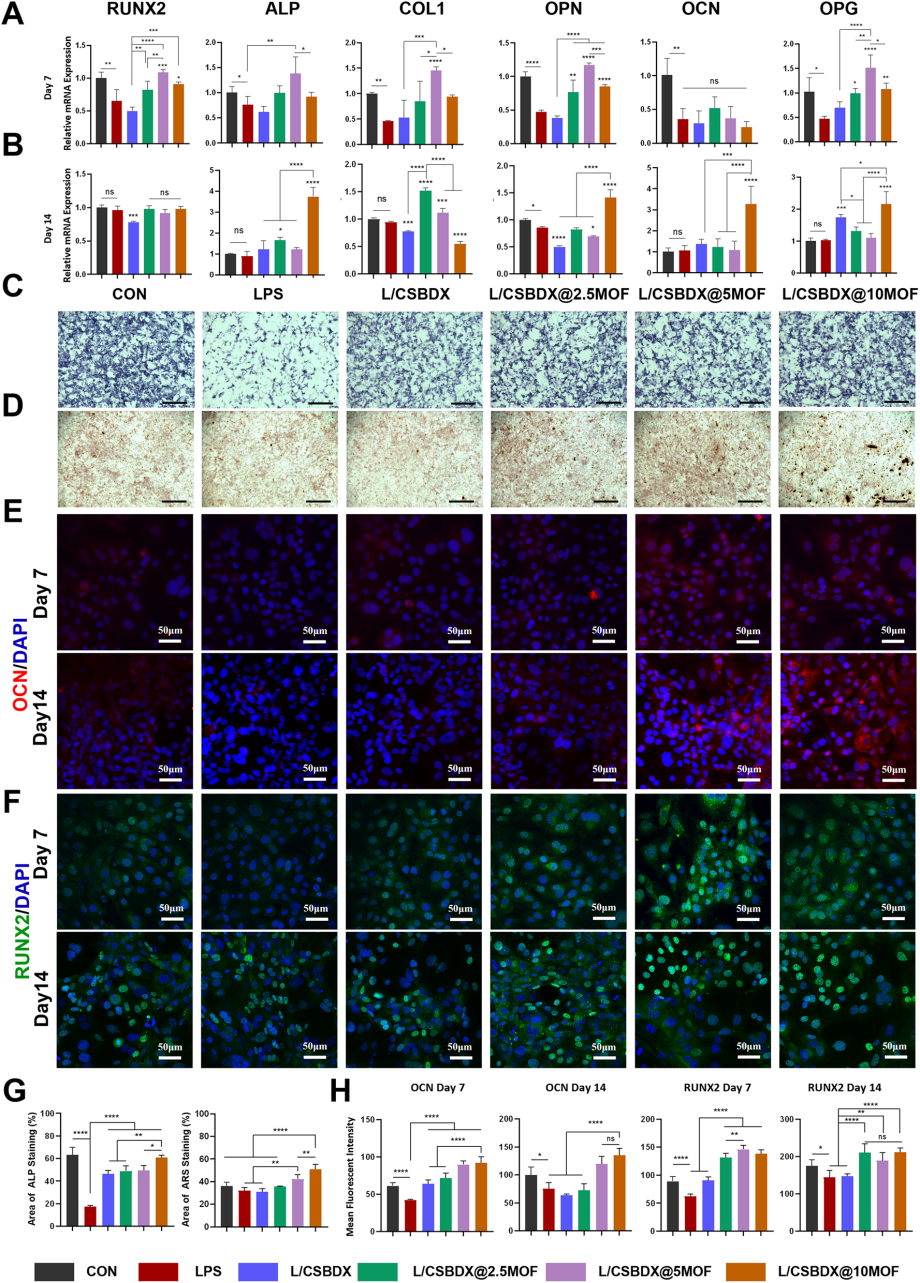
The osteogenic effect of CSBDX@MOF. Relative gene expression of RUNX2, ALP, COL1, OPN, OCN and OPG in MC3T3 cells incubated with macrophage conditioned medium on 7th day **A** and 14th day **B** (n = 4). **C** ALP staining of MC3T3 cells incubated with macrophage conditioned medium on 7th day (bar = 500 μm). **D** ARS staining of MC3T3 cells incubated with macrophage conditioned medium on 21st day (bar = 500 μm, n = 4). The representative immunofluorescence images of OCN (E) and RUNX2 (F) of MC3T3 cells incubated with macrophage conditioned medium on 7th and 14th day (bar = 50 μm). **G** The statistical analysis of area of ALP and ARS staining. **H** The statistical analysis of mean fluorescence intensity (MFI) of OCN and RUNX2 (n = 4). *: *P* < 0.05, **: *P* < 0.01, ***: *P* < 0.001, ****: *P* < 0.0001, *ns* no significance

At appropriate concentrations, Mg^2+^ has been demonstrated to effectively promote osteogenic differentiation [70, 71]. It has been reported that the high-molecular-weight polymer involving GA significantly enhances osteogenesis through the classical Wnt/β-catenin signaling pathway [72]. Furthermore, the combination of these two entities, termed Mg-GA, also exhibits pronounced in vitro osteogenic properties [73], yet there is limited documentation on its modulation of osteogenesis through immunoregulation. To validate osteogenic properties through immunoregulation, MC3T3 cells were subjected to osteogenic induction after a macrophage-conditioned medium culture. It is acknowledged that the expressions of Runt-related transcription factor 2 (RUNX2), alkaline phosphatase (ALP) and collagen 1 (COL1) are considered early indicators of osteogenesis, while the expressions of osteopontin (OPN), osteocalcin (OCN) and osteoprotegerin (OPG) represent relatively later osteogenic markers. After 7 days of cultivation, the mRNA expression levels of osteogenesis-related genes, including RUNX2, ALP, COL1, OPN and OPG were significantly upregulated in the CSBDX@MOF group, with the CSBDX@5MOF subgroup displaying the highest expression levels (Fig. 7A). On the 14th day, compared to the LPS group, CSBDX@10MOF exhibited continued significant upregulation in ALP, OPN, OCN and OPG mRNA expression (Fig. 7B). It is noteworthy that there was no significant difference between the CSBDX@MOF and LPS groups in the expression levels of OCN mRNA on day 7 and RUNX2 mRNA on day 14. ALP staining images mirrored the trends observed in qPCR results, with the CSBDX@5MOF group displaying the most distinct staining and the largest stained area (Fig. 7C, G). Immunofluorescence staining results demonstrated more optimal outcomes of CSBDX@5MOF group and CSBDX@10MOF group, for the OCN and RUNX2 expression of these two groups were significantly higher than that of LPS group. (Fig. 7E, F, H). After 21 days of osteogenic induction, noticeable calcium nodules were observed in the three CSBDX@MOF group (Fig. 7D, G). Osteogenesis is a dynamic long-term process, and the osteoimmunomodulatory effects of the hydrogel system vary in gene expression at different stages of osteogenesis. These outcomes may be attributed to the immunomodulatory effect of macrophage conditioned medium, for the concentration of free Mg^2+^, GA, and Mg-GA were relatively low after culture. The hydrogel system investigated in this study aims to create a favorable microenvironment for bone regeneration. Based on a comprehensive assessment of the aforementioned in vitro results, CSBDX@10MOF was selected for subsequent in vivo experiments.

### Alveolar bone regeneration in vivo through immunomodulation

**Fig. 8:**
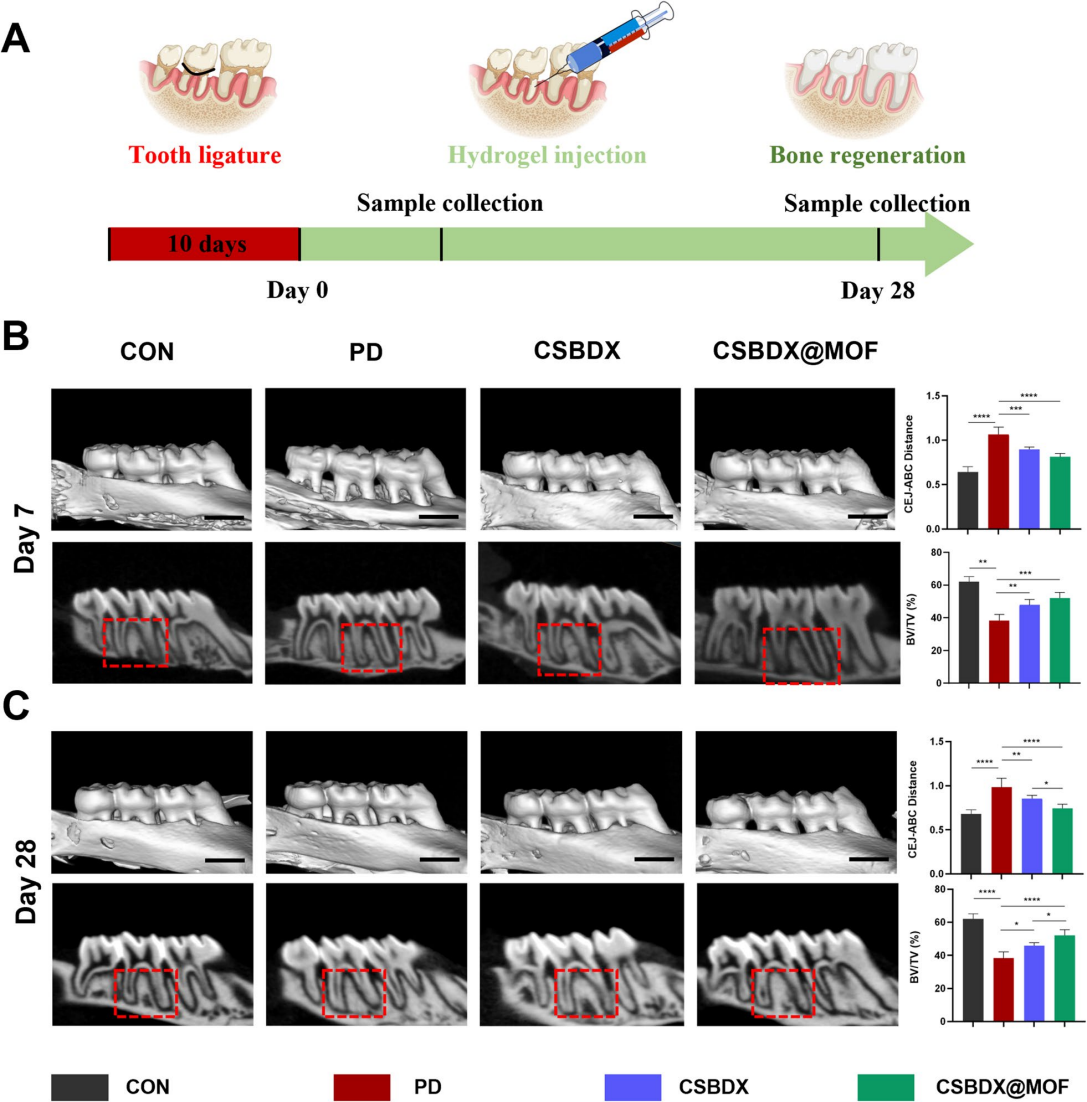
Evaluation of alveolar bone regeneration using micro-CT. **A** The schematic diagram of animal experiment. Representative micro-CT three-dimensional reconstruction and section images and CEJ-ABC distance, BV/TV quantitative analysis on the 7th day **B** and 28th day **C** (n = 6). *: *P* < 0.05, **: *P* < 0.01, ***: *P* < 0.001, ****: *P* < 0.0001, *ns* no significance

**Fig. 9:**
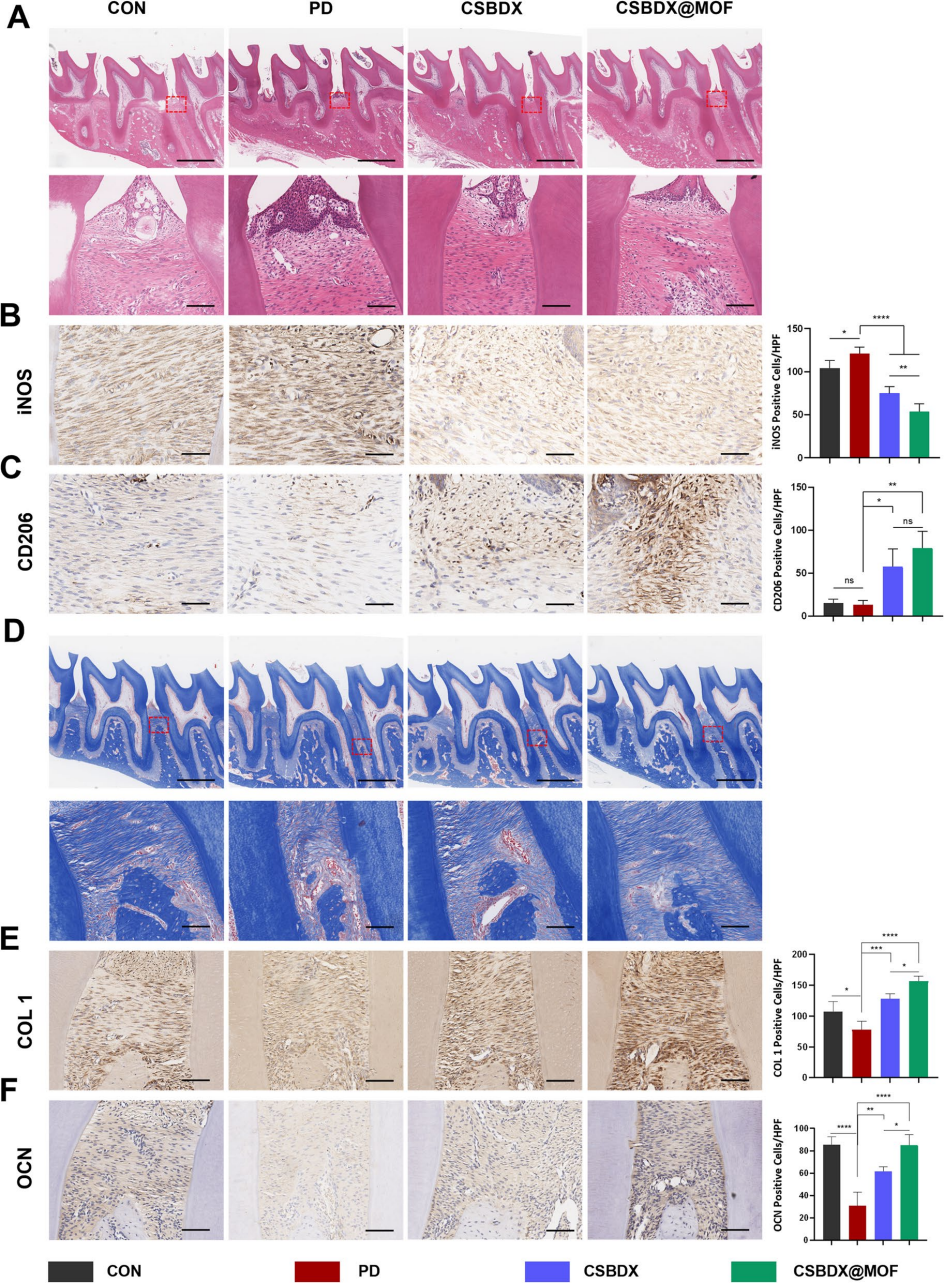
Histological evaluation of immunomodulatory, collagen deposit and alveolar bone regeneration of hydrogels. Representative H&E staining images on the 7th day **A** (bar = 1 mm upper, bar = 100 μm lower). Representative immunohistochemical staining images of iNOS **B** and CD206 **C** on the 7th day and the statistical analysis of positive cells of iNOS and CD206 per HPF (bar = 100 μm, n = 6). Representative Masson’s trichrome staining images **D** (bar = 1 mm upper, bar = 100 μm lower). Representative immunohistochemical staining images of COL1 **E** and OCN **F** on the 28th day and the statistical analysis of positive cells of COL 1 and OCN per HPF (bar = 100 μm, n = 6). *: *P* < 0.05, **: *P* < 0.01, ***: *P* < 0.001, ****: *P* < 0.0001, *ns* no significance

Periodontitis is often associated with alveolar bone destruction, and its inflammatory microenvironment directly inhibits bone regeneration. Here, a rat periodontitis model was established and used to evaluate the in vivo immunomodulatory effects and actual bone regeneration outcomes of a hydrogel system. Employing the classical ligature-induced method to induce periodontitis, the hydrogel system was administered into the periodontal pockets after model construction. On the 7th day, residual hydrogel can be observed. At this stage, we mainly focus on the inflammatory status of periodontal tissues and the expression of macrophage polarization markers. Due to the reparative function of our hydrogel system, along with the dosage at implantation and the timing of detection, by the 28th day, the hydrogel has almost completely disappeared, coinciding with periodontal tissue regeneration. Maxillary bone samples were collected on the 7th and 28th day (Fig. 8A), and Micro-CT was used to assess alveolar bone loss and regeneration. Three-dimensional reconstructed images showed no evident alveolar bone resorption in the CON group, whereas the periodontitis (PD) group exhibited substantial bone loss and exposed tooth roots, confirming the successful establishment of the periodontitis model. Compared to the PD group, CSBDX and CSBDX@MOF reversed alveolar bone loss, with the CSBDX@MOF group demonstrating increased alveolar bone mass on the 28th day. Measurements of the distance between the cementoenamel junction (CEJ) and the alveolar bone crest (ABC) were conducted to quantify the level of alveolar bone loss. The PD group had a significantly larger CEJ-ABC distance compared to the CON group, while the hydrogel-treated groups showed a significant reduction in CEJ-ABC distance at all time points (Fig. 8B, C). Notably, the CEJ-ABC distance in the CSBDX@MOF group was significantly shorter than that in the CSBDX group on the 28th day. Additionally, bone volume/total volume (BV/TV) ratio was employed to assess alveolar bone quality. The PD group exhibited the lowest BV/TV, whereas both CSBDX and CSBDX@MOF consistently displayed optimal outcomes. Remarkably, the BV/TV ratio of CSBDX@MOF was significantly higher than that in the CSBDX group on the 28th day. Consequently, the CSBDX@MOF group showed the most effective treatment for periodontitis-induced alveolar bone resorption. Hematoxylin and eosin (H&E) staining as well as Immunohistochemical (IHC) staining for iNOS and CD206 on the 7th day were performed to evaluate histological morphology of alveolar bone and immunomodulatory effects of the hydrogel system. As depicted in Fig. 9A, H&E staining supported the trends of micro-CT scanning. Besides, the PD group exhibited a high quantity of inflammatory cells, indicating the sustained presence of periodontitis features. In contrast, the hydrogel-treated groups showed a significant reduction in infiltrating inflammatory cells. For IHC staining, compared to the control group, the CSBDX@MOF group exhibited a significant reduction in iNOS-positive cells, while displaying the highest CD206 staining (Fig. 9B, C), highlighting its robust anti-inflammatory capabilities. Furthermore, Masson's trichrome staining and IHC staining for COL1 and OCN were conducted to evaluate periodontal collagen deposit and alveolar bone regeneration effects of the hydrogel system on the 28th day. Masson's trichrome staining revealed the presence of an organized periodontal ligament between the alveolar bone and tooth, accompanied by mature collagen formation in the CSBDX@MOF group **(**Fig. 9D). As depicted in Fig. 9E, F IHC staining revealed decreased expression levels of COL1 and OCN in the periodontitis environment, whereas the CSBDX and CSBDX@MOF groups showed more COL1 and OCN-positive cells, with the CSBDX@MOF group demonstrating superior effects. However, for periodontitis is a chronic disease and bone regeneration takes a long time, the relative long-term osteogenic effect of CSBDX and CSBDX@MOF system in vivo remains to be explored. In vitro studies have indicated the critical role of macrophage polarization in the onset and progression of periodontitis [67]. The aforementioned results validate that inhibiting M1 macrophage polarization can reduce the expression of inflammatory cytokines in periodontal lesion tissues [74], and inducing M2 macrophages can prevent bone loss [75, 76]. Therefore, CSBDX@MOF can promote alveolar bone regeneration by regulating M1/M2 polarization. As anticipated, the hydrogel system in this study demonstrated application efficacy in vivo by alleviating inflammation and promoting alveolar bone regeneration, attributed to the synergistic effects of CMCS’s antibacterial capabilities, 4-FPBA's reductive properties and DEX's anti-inflammatory effects. Particularly, the introduction of MOF indirectly provided Mg^2+^ and GA components, significantly improving the periodontal microenvironment, thereby imparting the hydrogel system with antioxidative capabilities and expediting bone formation by modulating macrophage polarization.

## Conclusion

In conclusion, periodontitis remains a significant challenge in dentistry due to its chronic inflammatory nature and the complexities associated with bone repair in the unique bacterial microenvironment. This study successfully introduced a novel therapeutic approach harnessing MOFs of Mg-GA to address alveolar bone defects. To overcome hurdles related to sustained drug release and the dynamic oral environment, a responsive hydrogel system was also devised. This smart hydrogel formed a dual-crosslinked network with the MOF (CSBDX@MOF), which is capable of on-demand release of therapeutic and bioactive components based on the heightened sensitivity to high pH and elevated levels of reactive oxygen species (ROS) of periodontitis, The study conducted in vivo and in vitro experiments validating the facile injectability, self-healing and biocompatibility of the CSBDX@MOF, as well as its efficacy in antibacterial functions, immune modulation and promotion of alveolar bone regeneration in periodontitis. The results further indicated that the precise control within the complex oral microenvironment represented a significant step toward tailored and effective therapies for periodontitis-induced bone defects. This innovative Dynamic Hydrogel-Metal–Organic Framework System exhibits promise as a potential therapeutic avenue for addressing the challenges in periodontitis treatment.

### Supplementary Information

Supplementary material 1.

## Data Availability

This published article includes all data generated and analyzed during this research.
