# Supplementary material for: Dynamic hydrogel–metal–organic framework system promotes bone regeneration in periodontitis through controlled drug delivery
Source: J Nanobiotechnology. 2024 May 26;22:287. doi: 10.1186/s12951-024-02555-9 (PMC11129436; doi:10.1186/s12951-024-02555-9)
Supplement: Supplementary file 1 — Supplementary material 1. [file 12951_2024_2555_MOESM1_ESM.docx]

**Supplementary material**


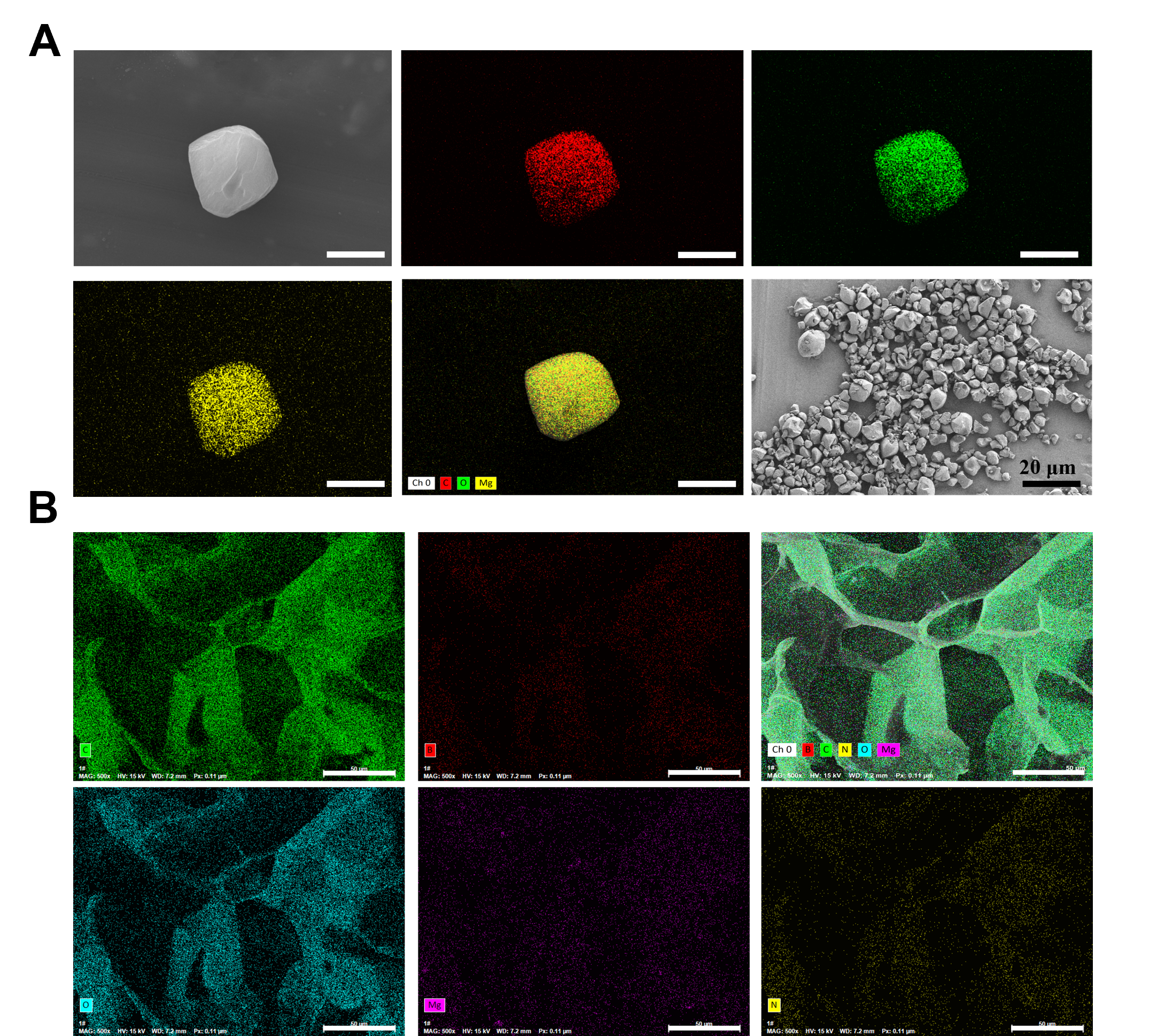


**Fig. S1.** The characterization of Mg-Ga and CSBDX@MOF. (A) The morphology and EDS mapping images of C, O and Mg element of Mg-GA (white bar = 2 μm, black bar = 20 μm). (B) morphology and EDS mapping images of C, O, B, N and Mg element of CSBDX@MOF (bar = 50 μm).


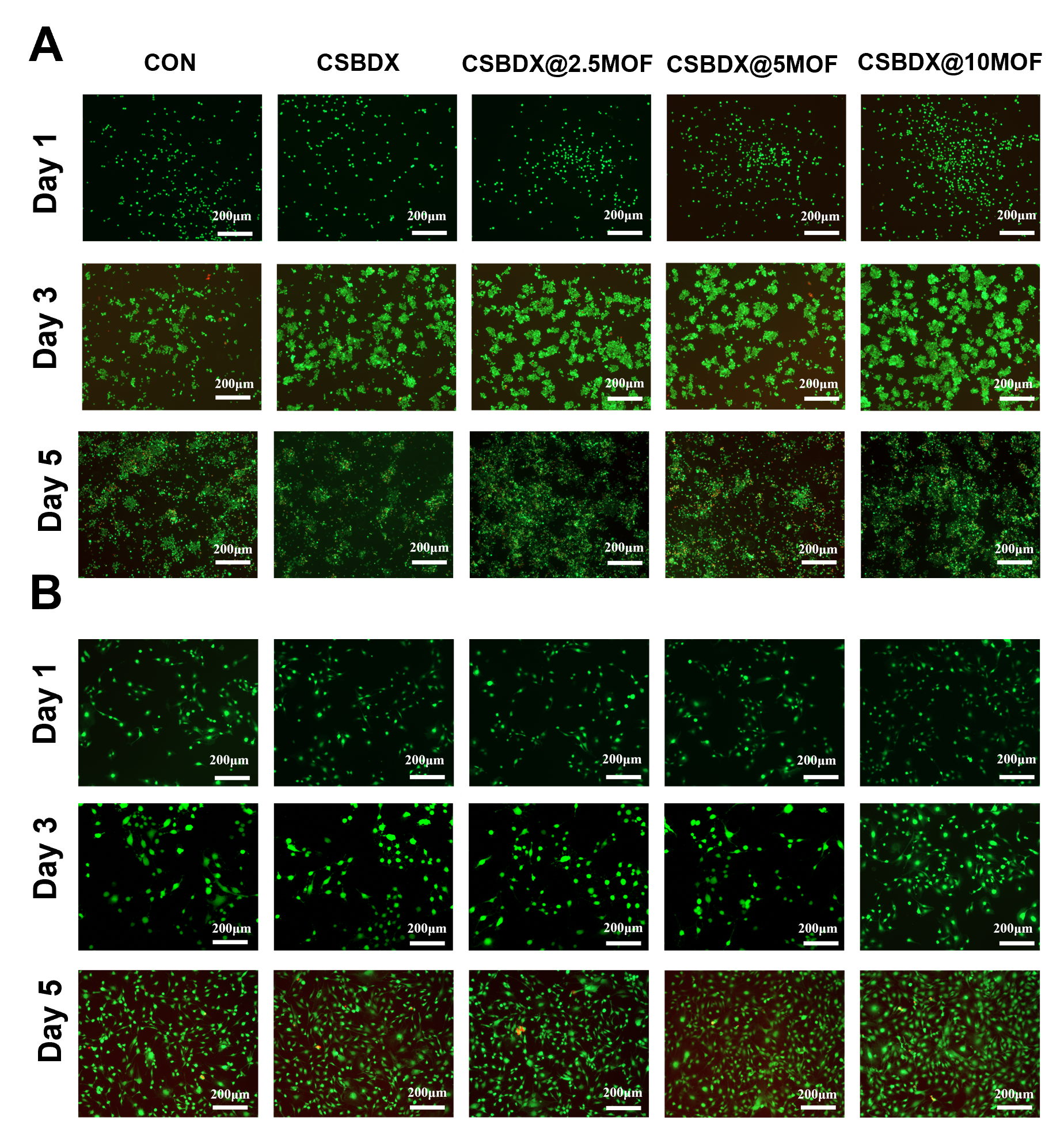


**Fig. S2.** The biocompatibility of CSBDX@MOF. The live/dead staining of RAW264.7s (A) and MC3T3 cells (B) cultured with hydrogel extracts for 1, 3 and 5 days (bar = 200 μm).

**Supplementary Table 1. Primers used for QRT-PCR**

| **Gene** | **Direction** | **Sequences** |
| --- | --- | --- |
| *iNOS* | Forward | 5’ CAG AAG TGC AAA GTC TCA GAC AT 3’ |
|  | Reverse | 5’ GTC ATC TTG TAT TGT TGG GCT 3’ |
| *COX-2* | Forward | 5’ AGA AGG AAA TGG CTG CAG AA 3’ |
|  | Reverse | 5’ GCT CGG CTT CCA GTA TTG AG 3’ |
| *IL-6* | Forward | 5’ AGT TGT GCA ATG GCA ATT CTG A 3’ |
|  | Reverse | 5’ AGG ACT CTG GCT TTG TCT TTC T 3’ |
| *TGF-β3* | Forward | 5’ CAA CAC CCT GAA CCC AGA G 3’ |
|  | Reverse | 5’ CTT CAC CAC CAT GTT GGA CAG 3’ |
| *IL-10* | Forward | 5’ TCC CTG GGT GAG AAG CTG AAG AC 3’ |
|  | Reverse | 5’ CAC CTG CTC CAC TGC CTT GC 3’ |
| *CD206* | Forward | 5’ AGA CGA AAT CCC TGC TAC TG 3’ |
|  | Reverse | 5’ CAC CCA TTC GAA GGC ATT C 3’ |
| *RUNX2* | Forward | 5’ AAG TGT TCT GTG GTC TCT GAG TTG A 3’ |
|  | Reverse | 5’ GCT GTA TGG TGA GGC TGG TAG G 3’ |
| *ALP* | Forward | 5’ TTG TAC GTC TTG GAG AGG GC 3’ |
|  | Reverse | 5’ TCA GAA GCT CAA CAC CAA CG3’ |
| *COL 1* | Forward | 5’ GGG TCT AGA CAT GTT CAG CTT TGT G 3’ |
|  | Reverse | 5’ ACC CTT AGG CCA TTG TGT ATG C 3’ |
| *OCN* | Forward | 5’ CAC TCC TCG CCC TAT TGG C 3’ |
|  | Reverse | 5’ CCC TCC TGC TTG GAC ACA AAG 3’ |
| *OPN* | Forward | 5’ CTC CAT TGA CTC GAA CGA CTC 3’ |
|  | Reverse | 5’ CAG GTC TGC GAA ACT TCT TAG AT 3’ |
| *OPG* | Forward | 5’ CAA AGG CAG GGC ATA CTT C 3’ |
|  | Reverse | 5’ TTC AAT GAT GTC CAA GAA CAC C3’ |
